# Selenium‐Containing Type‐I Organic Photosensitizers with Dual Reactive Oxygen Species of Superoxide and Hydroxyl Radicals as Switch‐Hitter for Photodynamic Therapy

**DOI:** 10.1002/advs.202301902

**Published:** 2023-06-25

**Authors:** Haiyang Wang, Tian Qin, Wen Wang, Xie Zhou, Faxu Lin, Guodong Liang, Zhiyong Yang, Zhenguo Chi, Ben Zhong Tang

**Affiliations:** ^1^ PCFM lab Guangdong Engineering Technology Research Center for High‐performance Organic and Polymer Photoelectric Functional Films School of Chemistry Sun Yat‐sen University Guangzhou 510275 P. R. China; ^2^ School of Pharmaceutical Sciences Sun Yat‐sen University Guangzhou 510275 P. R. China; ^3^ School of Materials Science and Engineering Sun Yat‐sen University Guangzhou 510275 P. R. China; ^4^ School of Science and Engineering Shenzhen Institute of Molecular Aggregate Science and Engineering the Chinese University of Hong Kong Shenzhen Guangdong 518172 P. R. China

**Keywords:** aggregation‐induced emission, hydroxyl radicals, selenium‐containing, superoxide radicals, type‐I photosensitizers

## Abstract

Organic type‐I photosensitizers (PSs) which produce aggressive reactive oxygen species (ROS) with less oxygen‐dependent exhibit attractive curative effect for photodynamic therapy (PDT), as they adapt better to hypoxia microenvironment in tumors. However, the reported type‐I PSs are limited and its exacted mechanism of oxygen dependence is still unclear. Herein, new selenium‐containing type‐I PSs of Se6 and Se5 with benzoselenadiazole acceptor has been designed and possessed aggregation‐induced emission characteristic. Benefited from double heavy‐atom‐effect of selenium and bromine, Se6 shows a smaller energy gap (Δ*E*_ST_) of 0.03 eV and improves ROS efficiency. Interestingly, type‐I radicals of both long‐lived superoxide anion (O_2_^•‾^) and short‐lived hydroxyl (^•^OH) are generated from them upon irradiation. This may provide a switch‐hitter of dual‐radical with complementary lifetimes for PDT. More importantly, simultaneous processes to produce ^•^OH are revealed, including disproportionation of O_2_^•‾^ and reaction between excited PS and water. Actually, Se6 displays superior in–vitro PDT performance to commercial chlorin e6 (Ce6), under normoxia or hypoxia. After intravenous injection, a significantly in–vivo PDT performance is demonstrated on Se6, where tumor growth inhibition rates of 99% is higher than Ce6. These findings offer new insights about both molecular design and mechanism study of type‐I PSs.

## Graphical abstract

By introducing double heavy‐atoms of Se and Br, a new type‐I photosensitizer of Se6 have been designed and exhibit high efficiencies of generating radicals of both O_2_^•‾^ and ^•^OH upon irradiation. This provides a switch‐hitter of dual‐radical with complementary lifetimes for outstanding photodynamic therapy in hypoxia, leading to significant anticancer outcome with tumor growth inhibition rates of 99%.

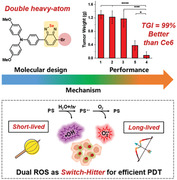

## 1 Introduction

Photodynamic therapy (PDT) has attracted intense attentions as a promising approach for oncology medicine, which possessed high spatiotemporal precision and noninvasion benefiting from its favorable controllability by visible or near infrared light.^[^ ^1^, ^2^, ^3^, ^4^ ^]^ PDT has been proven to be curative for several cancers such as skin cancer and continued treatment of eliminating the residuals after surgery of tumors.^[^ ^5^ ^]^ Upon light irradiation, excited photosensitizers (PSs) accumulated in tumor tissues generated reactive oxygen species (ROS) to induce cell apoptosis or necrosis, vascular damage, and probably immunoreaction for cancer treatment.^[^ ^6^ ^]^ A variety of PSs have been developed and exhibited excellent therapeutic responses toward tumors, undergoing different ROS generation through electron/proton transfer (type‐I) or energy transfer (type‐II) processes.^[^ ^7^, ^8^, ^9^, ^10^, ^11^ ^]^ Type‐II PSs transferred energy to ground state oxygen (^3^O_2_) and produced ROS of singlet oxygen (^1^O_2_), which thus suffered from strong O_2_ shortage of aberrant microenvironment in solid tumors.^[^ ^12^ ^]^ After excited, Type‐I PSs transferred electrons to the surrounding substrates and produced ROS specials such as superoxide anion (O_2_^•‾^)^[^ ^13^, ^14^, ^15^ ^]^ and hydroxyl^[^ ^16^, ^17^, ^18^ ^]^ (^•^OH) radicals. These radicals are less O_2_ dependent and areconsidered to be the more aggressive reactive ROS in biology.^[^ ^5^, ^19^, ^20^, ^21^ ^]^ Additionally, ^•^OH radical exhibits a very high oxidizing ability but possesses a short lifetime in nanoseconds.^[^ ^22^ ^]^ While O_2_^•‾^ radical with a very long lifetime of 1 s in aqueous solution has a lower oxidizing ability. Recently, several organic type‐I PSs have been designed and exhibited attractive curative effects for PDT, as they possessed obvious merits of well biosafety and structural diversity compared to the inorganic analogues.^[^ ^23^, ^24^, ^25^, ^26^ ^]^ However, different from the generation of two or more kinds of ROS simultaneously in most inorganic type‐I PSs, organic type‐I PSs produced two or more kinds of ROS were still limited.^[^ ^17^, ^18^, ^27^, ^28^ ^]^ On the other hand, the generation processes for O_2_^•‾^ and ^•^OH in type‐I PSs are complicated and controversial.^[^ ^22^, ^29^, ^30^ ^]^ In general, the excited triplet PSs could first transmit an electron to ^3^O_2_ to form O_2_^•‾^ specials, which could be further transferred to ^•^OH through superoxide disproportionation or Franck–Condon reaction, accompanied by the production of ^3^O_2_.^[^ ^31^, ^32^ ^]^ And it is also reported that ^•^OH also could be generated from reactions between excited triplet PSs and water in few references.^[^ ^33^ ^]^ Actually, the exact mechanism of ROS generation and their O_2_ dependent properties in detail are still unclear for organic type‐I PSs, which impeded the design of new organic molecules utilized as efficient PSs in PDT.

**Figure 1:**
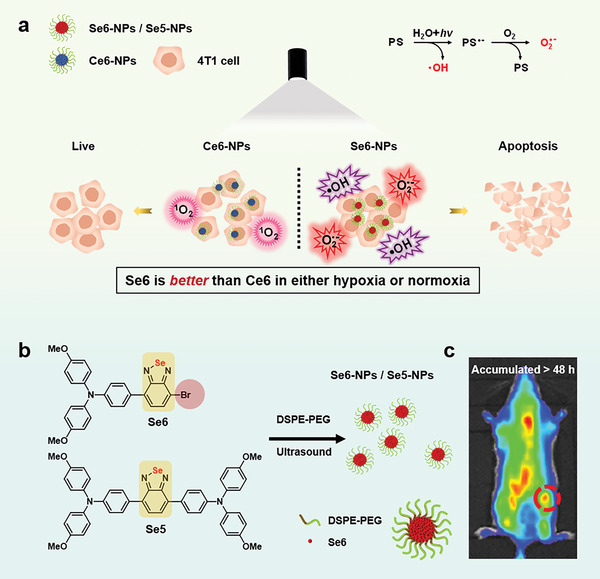
a) Illustration of photodynamic therapy (PDT) processes of type‐I Se6‐NPs and type‐II chlorin e6 (Ce6)‐NPs: Se6‐NPs generated dual radicals of ^•^OH and O_2_^•‾^ and exhibited better PDT treatment than Ce6‐NPs in either hypoxia or normoxia conditions. The proposed generated processes of radicals from Se6‐NPs in anaqueous solution were inset as well (top right). b) Molecular structures and nanoparticles of Se6 and Se5, the structure of Se6 nanoparticles was inset, andDSPE‐PEG is an amphiphilic copolymer. c) Se6‐NPs were accumulated in tumor sites more than 48 h after tail vein injection in tumor bearing mice.

Selenium (Se), as one of the essential trace elements in human body, has been proven to play important roles on in a wide range of biological processes.^[^ ^34^, ^35^, ^36^, ^37^ ^]^ And extensive reports about Se‐containing materials indicated the role of Se in cancer cell apoptosis with minimal side effects on normal cells.^[^ ^38^, ^39^, ^40^, ^41^, ^42^, ^43^ ^]^ Actually, the heavy‐atom effect of Se facilitated to enhance the intersystem crossing (ISC) rate for triplet excitons generation, which would improve the photodynamic effect.^[^ ^30^ ^]^ However, efficient PSs based on Se‐containing small molecules are limited. And strong electron acceptors containing Se, such as benzoselenadiazole (BSeD) and its derivatives, are seldom used to construct PSs for PDT applications, despite their excellent performance in designing near‐infrared (NIR) dyes for biological imaging.^[^ ^44^ ^]^ Herein, two efficient type‐I organic PSs based on Se‐containing molecules (Se6 and Se5) with aggregation‐induced emission (AIE) characteristics have been designed, which contained BSeD as acceptor and methoxyl‐triphenylamine as donor (**Figure** **1**). Especially for Se6, another heavy atom of bromine (Br) was also introduced into its molecular structure, in order to further enhance its ISC efficiency. Benefited from the double heavy‐atom‐effect of Se and Br plus strong intramolecular charge transfer (ICT) properties, they possessed high ISC rate, yielding abundant triplet excitons for ROS generation. Interestingly, these compounds can generate type‐I radicals of both O_2_^•‾^ and ^•^OH but no type‐II radical of ^1^O_2_ after irradiated by white light. Because of the complementary radical lifetimes of these two type‐I radicals, these newly designed PSs thus might provide a switch‐hitter for outstanding PDT. Even more surprisingly, both two processes to produce ^•^OH radicals with or without O_2_ participation, either from superoxide disproportionation of O_2_^•‾^ or from excited PS reacted with water, could be simultaneously revealed in the systems. Therefore, the new PSs of Se6 and Se5 were proved to produce ^•^OH efficiently without any oxygen, obtaining a complete remission of oxygen for PDT in the hypoxic environment of tumors. Actually, in vitro results validated that Se6 with double heavy atoms performed better therapeutic effect than that of commercial PS of chlorin e6 (Ce6) in PDT treatment of 4T1 cells. And in vivo experimental data on 4T1‐tumor‐bearing mice by intravenous injection further demonstrated that Se6 exhibited a significant PDT performance, with anticancer outcome of tumor growth inhibition (TGI) rates of 99% and higher than Ce6 as well.

## 2 Result and Discussion

In recent years, many donor–acceptor (D–A) type organic compounds with benzothiadiazole as acceptor have exhibited impressive performance on biological imaging and PDT, profiting from its strong electron withdrawal ability. A few reports about NIR dyes containing anacceptor of BSeD with alternative Se for S, indicated that BSeD showed astronger ability of electron withdrawing.^[^ ^44^ ^]^ As the heavy atom effect of Se, molecules and polymers containing Se usually exhibited high ISC rate for generating triplet excitons, improving the ROS efficiency of PSs. In this report, BSeD was chosen as acceptor to design new D–A type organic PSs, which was expected to integrate strong ICT and heavy atom effect into the new compounds. Additionally, bi‐(4‐methoxyl)‐triphenylamine was used as donor, which provided not only astrong ability of electron donating but also twist conformation for preventing strong *π*–*π* quenching in solid state. Two new organic PSs, namely Se6 and Se5, were designed and studied for their PDT effect. Especially, another heavy atom of Br was also introduced into Se6 to further enhance its ISC efficiency. For comparison, an analogue (SeH) of Se6 without Br substitution was also studied. As shown in Schemes S1 and S2, Supporting Information, Se6, Se5, and SeH were synthesized by coupling bi‐(4‐methoxyl)‐triphenylamine and BSeD. Additionally, a reported compound (S5) with benzothiadiazole acceptor, was also synthesized to compare with Se5. Their chemical structures were confirmed carefully by nuclear magnetic resonance and high‐resolution mass spectrometry with high purity (Figures S24–S32, Supporting Information).

**Figure 2:**
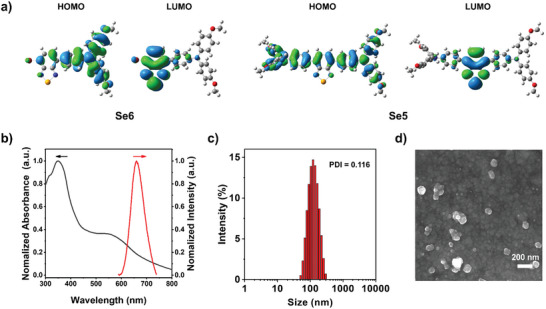
a) The DFT calculated electron cloud distributions in HOMOs and LUMOs of Se6 and Se5. b) Normalized absorption and fluorescence spectra of Se6‐NPs in aqueous solution (10 µm). c) The particle size distribution of Se6‐NPs detected from dynamic light scattering (DLS). d) Scanning electron microscopy (SEM) images of Se6‐NPs, scale bar: 200 nm. The polydispersity index (PDI) of nanoparticles was also inset in (c).

The optical properties of these PSs were investigated by UV–vis and photoluminescence (PL) spectroscopy. The typical charge transfer (CT) type broad peaks in their UV–vis absorption spectra in solutions were in the range of 400–600 nm, as shown in Figure S1, Supporting Information. The position of CT type peaks was similar for Se6, Se5, and S5, while SeH possessed a blue‐shifted absorption peak. Especially for Se6 and Se5, these peaks were centered at the same wavelength of 483 nm with an onset at 570 nm (equal to 2.18 eV). Their energy levels of highest occupied molecular orbitals (HOMOs) could be calculated from the cyclic voltammetry (CV) experiments, which were −5.24 and −5.08 eV for Se6 and Se5, respectively (Figure S2, Supporting Information). Combination of above results, the energy levels of lowest unoccupied molecular orbitals (LUMOs) could be also obtained for Se6 and Se5 as −3.06 and −2.90 eV, respectively. Se6 thus possessed both lower HOMO and LUMO than those of Se5, indicating capability of stronger electron‐accepting in LUMO but weaker electron‐donating in HOMO for Se6. These results were in good accordance with the density functional theory (DFT) calculated HOMOs and LUMOs, in which both HOMO and LUMO of Se6 were also lower than those of Se5 (Table S1, Supporting Information). The DFT calculated electron clouds of Se6 and Se5 exhibited clear separation between their HOMOs and LUMOs, which led to strong ICT excited states and were consistent with their broad UV–vis absorption peaks, as shown in **Figure** **2**a. The small overlap between HOMO and LUMO could reduce the energy gap (Δ*E*_ST_) between the lowest singlet excited (S_1_) state and the lowest triplet excited (T_1_) state. Actually, the experimental Δ*E*_ST_ of the two selenium‐containing molecules, Se6 and Se5, were as small as 0.03 and 0.07 eV, respectively, which could be calculated from the onsets of their fluorescence and phosphorescence spectra (Figure 2b and Figures S3 and S5a, Supporting Information). As containing double heavy atoms of Se and Br, Se6 actually possessed a smaller Δ*E*_ST_. Such small Δ*E*_ST_ could promote the ISC transition and subsequent the generation of triplet excitons, improving their ROS efficiency. Interestingly, both Se6 and Se5 showed integrated properties of ICT and AIE, revealed from their PL measurements on mixture of tetrahydrofuran (THF) and water.^[^ ^2^, ^6^ ^]^ As shown in Figure S4, Supporting Information, their PL intensities quenched in THF/water mixture along the water fraction increased to 60% due to their ICT characteristic. However, the fluorescence arose sharply when the water fraction further increased and surpassed 60%, unambiguously confirming their AIE features at aggregated state.

In order to endow Se6 or Se5 molecules with water solubility and subsequent stability in blood, nanoparticles (NPs) of Se6 or Se5 wrapped up by an amphiphilic copolymer, namely DSPE‐PEG2000, were fabricated through a nanoprecipitation method. The resulted Se6‐NPs and Se5‐NPs possessed an average hydrodynamic diameter of 115 and 87 nm in water revealed from the dynamic light scattering detection (Figure 2c and Figure S5b, Supporting Information). Their spherical nanostructures were confirmed by scanning electron microscope, as shown in Figure 2d and Figure S5c, Supporting Information. Compared to isolated molecules in solution state, the yield NPs, Se6‐NPs, and Se5‐NPs, exhibited redshifted absorption peaks around 550 and 518 nm, respectively, due to the *π*–*π* interactions in their aggregates (Figure 2b and Figure S5a, Supporting Information).

**Figure 3:**
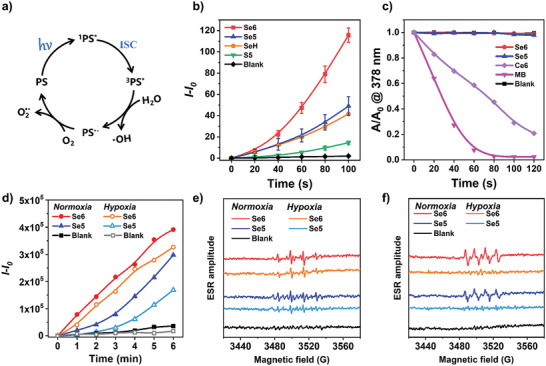
a) Proposed generation processes of hydroxyl and superoxide anion radicals. b) Relative changes on photoluminescence (PL) intensity of overall ROS probe DCFH versus irradiation time of white light in the presence of different PSs: Se6, Se5, SeH, and S5 (*n* = 3, mean ± SD). c) Decomposition rates of singlet oxygen probe ABDA upon irradiation of white light in the presence of Se6, Se5, Ce6, and MB. d) Relative PL intensity of hydroxyl radical probe HPF versus irradiation time of white light in the presence of Se6 or Se5. ESR signals of DMPO for e) hydroxyl radical and f) superoxide anion characterization in the presence of Se6 or Se5 (100 µm), before and after white light irradiation (100 mW cm^−2^). For probe detections, white light power was 20 mW cm^−2^, while the concentrations of PSs and probes were as follow: 30 µm for PSs (MB, Ce6, Se6, Se5, SeH, or S5), 25 µm for DCFH, 100 µm for ABDA, 20 µm for HPF, respectively. For the measurement of Ce6, the solution was a mixture of dimethylsulfoxide (DMSO) and water (1/1 V/V).

The overall ROS production efficiencies of these newly designed PSs were investigated by using a classic ROS indicator dichlorofluorescein (DCFH), in order to evaluate their PDT properties. The green fluorescence of DCFH can be sensitively activated by any type of ROS, thus its emission intensity indicates the overall amount of produced ROS in the system. As shown in **Figure** **3**b and Figure S6, Supporting Information, the fluorescence intensity of DCFH increased rapidly in the presence of each new PS, with the continuous irradiation of a weak white light (20 mW cm^−2^). In contrast, anegligible increase in fluorescence was detected for the irradiated solution of DCFH alone without any PSs. Notably, the increase in fluorescence of DCFH in the presence of Se6 kept higher than that in the case of Se5 or SeH upon continuous irradiation of white light, revealing superior overall ROS production efficiency for Se6 with two heavy atoms in its molecular structure. This result is in accordance with the experimental smaller Δ*E*_ST_ of Se6 (0.03 eV) and the theoretically more possible ISC channels in Se6, both leading to better ISC efficiency of Se6 (Figure S7, Supporting Information). Regarding to the difference of Se and S atoms, it was obvious that Se5 exhibited a bigger increase in fluorescence than S5, revealing the advantage of the Se containing group of BSeD for designing efficient PSs. This is also indicated by the smaller Δ*E*_ST_ of Se5 than that of S5 in the DFT calculated results (Figure S7b,d, Supporting Information).

In order to distinguish the ROS type, different ROS indicators beyond DCFH were employed. Initially, anindicator of 9,10‐anthracenediyl‐bis(methylene) dimalonic acid (ABDA) was selected to evaluate singlet oxygen (^1^O_2_) generation in the NPs. Nearly no apparent signal change in the absorption of ABDA was found in the systems containing Se6‐ or Se5‐NPs, as shown in Figure 3c and Figures S8 and S9, Supporting Information. Only the system in the presence of methyl blue (MB) or Ce6,^[^ ^45^ ^]^ both well‐known typical type‐II PSs, exhibited a sharp decrease of BADA absorption upon continuous irradiation of white light, implying their excellent capacity of ^1^O_2_ production. Therefore, compounds of Se6 and Se5 could not generate ^1^O_2_ and subsequently were not type‐II PSs as well. Then fluorescence indicator of dihydrorhodamine 123 (DHR123) was utilized to test whether type‐I ROS of O_2_^•‾^ could be generated by PSs of Se6 and Se5 upon irradiation. It was interesting to obtain an obvious enhancement of the DHR123 fluorescence of systems with Se6‐ or Se5‐NPs after white light irradiation (Figure S10, Supporting Information), which indicated the designed PSs could generate type‐I ROS of O_2_^•‾^. And the production of O_2_^•‾^ from Se6 and Se5 by light irradiation were further confirmed by electron spin resonance (ESR) measurements, by using 5,5‐dimethyl‐1‐pyrroline‐*N*‐oxide (DMPO) as the spin‐trap agent (Figure 3f). It is reported that radicals of O_2_^•‾^ can partially reproduce O_2_ from several complex reactions in vivo, which can reduce its dependence on O_2_. Unfortunately, the generation of O_2_^•‾^ radicals needs the help of O_2_, which makes PSs still suffer from the O_2_ shortage of microenvironment in tumors. Another type‐I ROS of ^•^OH was also inspected using the fluorescence indicator of hydroxyphenyl fluorescein (HPF), which can take reaction with ^•^OH to produce a green fluorescence. As shown in Figure S11, Supporting Information, the control sample was almost nonemissive after irradiation by white light in 6 min. Amazingly, the fluorescence enhanced obviously with the increasing time of irradiation for the sample with the presence of Se6‐ or Se5‐NPs (Figure 3d and Figure S11, Supporting Information), suggesting that both designed PSs were capable of generating ^•^OH upon irradiation of white light. The ESR measurement was also carried out to confirm the generation of ^•^OH, using the spin‐trap agent of DMPO. As clearly shown in Figure 3e, the four‐line ESR signals with anintensity ratio of about 1:2:2:1 were observed in the ESR spectrum of thesample with Se6‐ or Se5‐NPs after irradiation of white light, which could be attributed to the adducts of DMPO‐^•^OH and thus indicate the production of ^•^OH radicals. Comparing Se6 and Se5, Se6 with double heavy atoms of Se and Br possessed higher efficiency inbothO_2_^•‾^ and ^•^OH generation. Except for the more population of excited triplet PS enhanced by smaller Δ*E*_ST_, it would be also due to the lower energy level and subsequent stronger electron‐accepting ability of LUMO in Se6. These NPs also exhibited excellent stability during long‐time irradiation with white light, which indicated their potential application in PDT (Figure S12, Supporting Information).

It is generally known that type‐I PSs produced ^•^OH radicals through an electron/proton transfer process, in which oxygen played aminor role. However, the exact role of oxygen in the generation process of ^•^OH radicals is complex and still unclear.^[^ ^1^, ^6^ ^]^ In order to obtain a deep insight into the mechanism and its O_2_‐dependent property, the difference of ^•^OH generation in normoxia and hypoxia conditions has been studied. As shown in Figure 3d and Figure S11, Supporting Information, for both Se6 and Se5, they can generate plenty of ^•^OH radicals neither in normoxia or hypoxia conditions, as the fluorescence intensity at 515 nm still enhanced fast upon irradiation of white light even in the hypoxia condition. Especially for Se6, the fluorescence intensity only decreased about 20% in a hypoxia condition in comparison with that in a normoxia condition (Figure 3d). And the ESR signals also became a bit weaker in a hypoxia condition, consistent with the results detected by HPF (Figure 3e). This solidly revealed that type‐I radical of ^•^OH could be efficiently generated upon irradiation even in hypoxia condition such as microenvironment of tumors, implying its less O_2_ dependence. On the other hand, it also indicated these ^•^OH radicals were produced through two different processes at least. Most radicals were generated by a process without the participation of O_2_ but a minor part of ^•^OH radicals came from a process with the help of O_2_. As illustrated in Figure 3a, type‐I PSs could be able to transfer electrons to the surrounding O_2_ and generate O_2_^•‾^ simultaneously, which was proposed in many reported type‐I PS systems.^[^ ^31^, ^32^ ^]^ And it is well‐recognized that the O_2_^•‾^ radicals could further transform into ^•^OH radicals via multi‐step subsequent reactions. Therefore, the minor part of ^•^OH radicals should be from the transform of O_2_^•‾^ radicals. How about the most population of ^•^OH radicals, which could be generated without the assistance of O_2_ in hypoxia conditions? As only water and PSs in the system except HPF, it is reasonable that most ^•^OH radicals should be generated from reactions between excited triplet PS molecules and water molecules upon white light irradiation, which isalso proposed in some references.^[^ ^33^ ^]^ These results thus provided a comprehensive understanding of the O_2_ dependence property of ^•^OH radical, namely, two controversial hypothetical processes of ^•^OH generation may occur simultaneouslyin aqueous solution.

**Figure 4:**
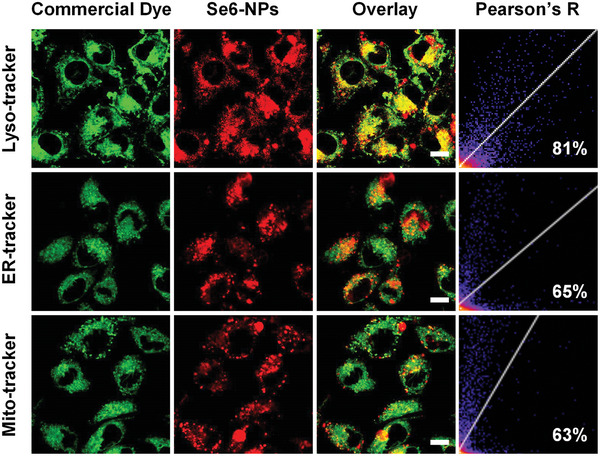
Colocalization images of 4T1 cells co‐stained with Se6‐NPs and different commercial dyes, including Lyso‐Tracker Green, ER‐Tracker Green, and Mito‐Tracker Green. The excitation (Ex) and emission (Em) wavelengths for commercial dyes: Ex = 488 nm, Em = 510–540 nm; for Se6‐NPs: Ex = 488 nm, Em = 650–690 nm; scale bar: 10 µm.

In view of the excellent type‐I ROS generation of dual radicals with complementary lifetimes, their potential biological applications were investigated. PDT was already confirmed to be an effectively clinical treatment for chest wall recurrences in selected patients.^[^ ^46^, ^47^ ^]^ Thus, many newly designed PSs were investigated for their curative effect on breast cancer, such as 4T1 cells, using PDT technique.^[^ ^1^, ^2^ ^]^ For easy comparison, the PDT effects on breast cancer cells (4T1) of the two type‐I PSs were also studied here. Initially, the endocytosis of these NPs into 4T1 cells had been tracked by confocal laser microscopy (CLSM) and the results were depicted in Figures S13 and S14, Supporting Information. It was found the occurrence of weak red fluorescence from these NPs inside 4T1 cells with a short incubation of 1 h, indicating the quick endocytosis of NPs. As incubation time extended to 8 h, the red fluorescence became much stronger, demonstrating the accumulation of Se6‐ or Se5‐NPs in 4T1 cells. The subcellular distribution profiles of Se6‐ and Se5‐NPs were then carefully assessed by colocalization analysis with several commercially available bioprobes, including Lyso‐Tracker Green for lysosome, ER‐Tracker Green for endoplasmic reticulum, and Mito‐Tracker Green for mitochondria. The colocalization images for Se6‐ and Se5‐NPs are shown in **Figure** **4** and Figure S15, Supporting Information, respectively. It is demonstrated that both Se6‐ and Se5‐NPs mainly accumulated in lysosomes, as evidenced by the high overlay of fluorescence between PSs and Lyso‐Tracker Green. Taking the red fluorescence of Se6‐NPs as an example, higher Pearson correlation coefficient of 85% was calculated for its overlay with the green fluorescence of Lyso‐Tracker Green, while only 65% and 63% for the fluorescence of ER‐Tracker Green and Mito‐Tracker Green, respectively.

**Figure 5:**
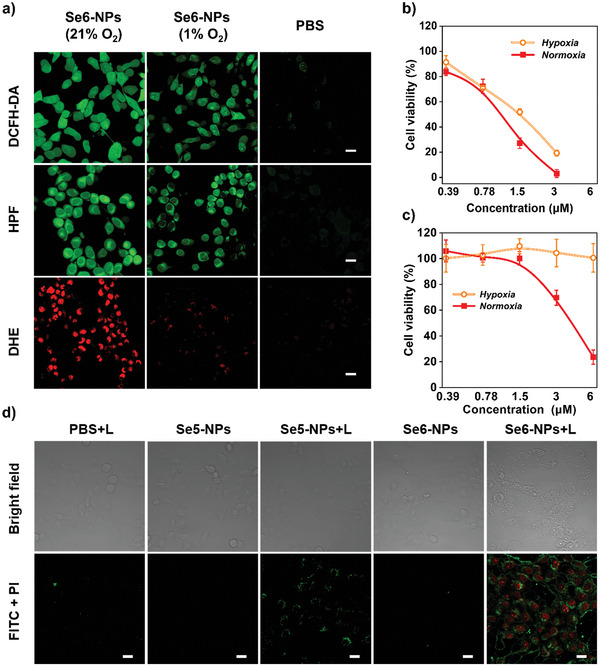
In vitro characterization of Se6‐NPs. a) Intracellular ROS generation by Se6‐NPs upon white light irradiation inside 4T1 cells: DCFH‐DA for overall ROS, HPF for hydroxyl radical, and DHE for superoxide anion. The PBS groups were treated by the same condition at normoxia but without Se6‐NPs, the excitation (Ex) and emission (Em) wavelengths for sensors: Ex = 488 nm, Em = 510–540 nm for DCFH‐DA, Em = 510–540 nm for HPF, Em = 580–620 nm for DHE, scale bar: 20 µm. Viability of 4T1 cells incubated with b) Se6‐NPs and c) Ce6‐NPs at different concentrations upon irradiation of 20 mW cm^2^ for 30 min under normoxia or hypoxia conditions (*n* = 6, mean ± SD). d) Apoptosis analysis using CLSM toward 4T1 cells in various groups after different treatments with or without light (L) irradiation. The excitation (Ex) and emission (Em) wavelengths for apoptosis kit (annexin V‐FITC/PI): Ex = 488 nm, Em = 500–530 nm for FITC, Em = 600–620 nm for PI, scale bar: 20 µm.

Subsequently, the light‐triggered ROS generation of Se6‐NPs inside 4T1 cells was also estimated by CLSM upon exposure to irradiation of white light. The safety of irradiation on 4T1 cells was confirmed before the experiments (Figure S16d, Supporting Information). And different fluorescence indicators had been used under both normoxia and hypoxia conditions, in order to deeply understand the cellular activities of this newly designed PS with dual radicals. After irradiation, astrongly green 2′,7′‐dichlorodihydrofluorescein diacetate (DCFH‐DA) fluorescence signal was distinctly observed in 4T1 cells, under either normoxia or hypoxia conditions, as can be seen from **Figure** **5**a. This revealed that Se6‐NPs could efficiently produce intracellular ROS regardless of oxygen concentration, which was consistent with the results of Se6‐NPs in anaqueous solution. Moreover, the intracellular generation of O_2_^•‾^ and ^•^OH were also studied by using indicators of dihydroethidium (DHE) and HPF, respectively, under both normoxia and hypoxia conditions. Interestingly, similar results about the response to oxygen concentration of O_2_^•‾^ and ^•^OH radicals generation inside cells were obtained from CLSM, compared with these in aqueous solution (Figure 5a). The red fluorescence of DHE inside 4T1 cells under normoxia condition would quench when the cells under hypoxia condition, indicating the strong oxygen dependence of intracellular O_2_^•‾^ generation. In contrast, thestrongly green fluorescence of HPF could be observed inside cells under both normoxia and hypoxia conditions, confirming that intracellular ^•^OH could efficiently generate regardless of high or low oxygen concentration. The green fluorescence became a little weaker but still strong under hypoxia condition, which also indicated that a minor part of ^•^OH radicals came from reactions relative to oxygen.

Before evaluating the photodynamic antitumor effect of Se6‐ and Se5‐NPs, their dark cytotoxicity to 4T1 cells was studied first. As shown in Figure S16a,b, Supporting Information, over 80% 4T1 cells were alive after incubation even in a high concentration of 25 µm of Se6‐ or Se5‐NPs, under either normoxia or hypoxia conditions, manifesting negligible dark cytotoxicity or excellent biocompatibility for both Se6‐ and Se5‐NPs. When irradiation with white light, phototoxicity against 4T1 cells were clearly observed for both Se6‐ and Se5‐NPs, under either high or low oxygen concentration conditions. At a very low concentration of NPs (3 µm) under normoxia condition, only 3.1% cells were still alive after irradiation in the presence of Se6‐NPs, which was about 69.1% for Se5‐NPs at the same condition (Figure 5b and Figure S16c, Supporting Information). Regarding to hypoxia condition, both NPs still exhibited excellent photodynamic tumoricidal efficacy which was similar to these under normoxia condition, implying their less dependence on oxygen for potential practical PDT treatment of tumors. It is noted that only 19.3% survival cells remained after irradiation with white light (20 mW cm^−2^) in the presence of a very low concentration of Se6‐NPs (3 µm) under hypoxia condition, which was among the best results reported of organic type‐I PSs. In addition, Se6 showed superior ROS generation capacity when compared to the popular commercially available PSs of Ce6. As plotted in Figure 5b,c, under the same white light irradiation and normoxia condition, Ce6 exhibited phototoxicity of 69.7% viability of 4T1 cells at the same concentration (3 µm) and reached similar viability (23.5%) at a higher concentration of 6 µm, which possessed much weaker phototoxicity than Se6. The ROS generation of Ce6 had been carefully investigated. It possesses a hydrophobic large planar structure and iseasily stacked in its nanoaggregates in anaqueous solution, which are revealed from the broadening and red‐shifting of its absorption peaks at the range of 500–700 nm (Figure S17, Supporting Information). As shown in Figure 3c and Figure S9, Supporting Information, Ce6 produced plenty of ROS (^1^O_2_) only in the mixture of dimethylsulfoxide (DMSO) and water (1/1 V/V) but much less quantity of ROS in either water or DMSO. This is because of the quenching by the formation of Ce6 stacking in water or small dissolved oxygen in DMSO. Therefore, Ce6, a type‐II PS, would show no any phototoxicity and leave 100% 4T1 cells alive under a hypoxia condition with 1% oxygen in anaqueous solution (PBS). These results further confirmed the outstanding efficient phototoxicity of our newly designed PSs, which possessed AIE properties and subsequently restrained the quenching of ROS generation in their nanoaggregates.^[^ ^6^, ^48^ ^]^

The cell apoptosis induced by the new type‐I PSs was further validated by CLSM and flow cytometry analysis using annexin V‐FITC/PI (fluorescein isothiocyanate/propidium iodide) stain method, as shown in Figure 5d and Figure S18, Supporting Information. It turned out that there were very small cell apoptosis occurrences before irradiation with white light in the presence of Se6‐ or Se5‐NPs in comparison with the control sample. On the contrast, a significant fluorescence presented for cells apoptosis could be observed after treatment with Se6‐ or Se5‐NPs plus light irradiation, while a high proportion of cells presented apoptosis was calculated to be 23% for Se5‐NPs and as high as 68% for Se6 NPs from flow cytometry analysis (Figure S18, Supporting Information). Additionally, except forthe damages to lysosome where these NPs were located in, damages to mitochondria were also confirmed by using JC‐1 mitochondrial membrane potential probe (Figure S19, Supporting Information). This is because of that the photodynamic treatment could increase the permeability of lysosomal membranes. Then ROS could enter the cytoplasm and subsequently cause damage to mitochondria. In order to check their applicability, the phototoxicities against RM‐1 cells of both Se6‐ and Se5‐NPs were investigated. As cells viability shown in Figure S20, Supporting Information, results similar to those for 4T1 cells were clearly observed, when irradiation with white light under either normoxia or hypoxia conditions. Therefore, it is confirmed that these new PSs with excellent photodynamic effects could be efficiently applied to other cancers.

**Figure 6:**
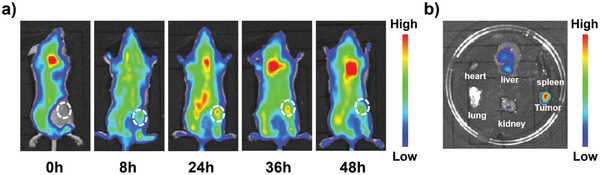
a) In vivo fluorescence images of Se6‐NPs distribution in the tumor‐bearing mice at different time after tail vein injection of Se6‐NPs. b) Ex vivo fluorescence images of main organs of tumor‐bearing mice at 48 h after tail vein injection. Wavelengths for excitation and emission: 520 and 670 nm, respectively. Concentration of Se6‐NPs: 100 µm in 100 µL.

Encouraged by the excellent intracellular PDT efficiency, the PDT capability of Se6 in vivo had been assessed on the 4T1‐tumor‐bearing BALB/c mice. The distribution and pharmacokinetics of Se6‐NPs in vivo were traced by fluorescence imaging utilizing the signals of Se6 itself, benefiting from its AIE property and obvious fluorescence in aggregated state. After intravenous injection on tail for about 8 h, obvious fluorescence of Se6 was detected at the tumor site, implying the quick accumulation of Se6‐NPs into tumor tissues due to the enhanced permeability and retention effect of solid tumor. The fluorescence in tumor location reached itsbrightest at ≈24 h and could keep at a high intensity and without a reduction at 36 h post‐injection, as clearly shown in **Figure** **6**a. Even after 48 h to injection, the fluorescence in tumor tissue was still significantly stronger than any other organs in the ex vivo imaging results, revealing the effective accumulation of Se6‐NPs in tumors (Figure 6b). Thus the times of administration of Se6‐NPs can be reduced, which could realize multiple irradiations at every administration and subsequently reduced the pain of injection. In addition, almost no fluorescence signals of Se6‐NPs could be detected in blood of treated mice after 48 h, indicating a half‐life period of 11.3 h (Figure S21a, Supporting Information). And only some fluorescence was seen in liver among major organs, which also demonstrated that Se6‐NPs could be eliminated and safe for in vivo application (Figure S21b,c, Supporting Information).

**Figure 7:**
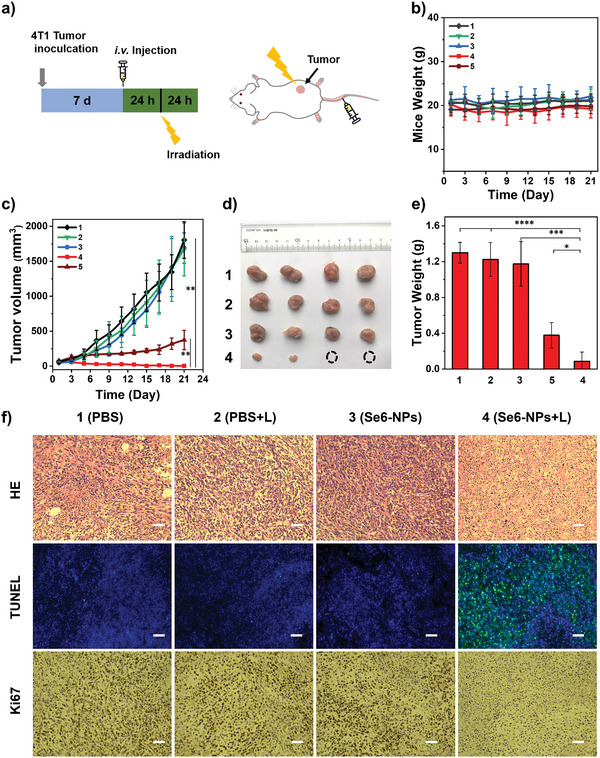
In vivo PDT treatment characteristic of Se6‐NPs on 4T1‐tumor‐bearing mice. a) Schematic diagram of the PDT treatment process with Se6‐NPs/Ce6‐NPs: intravenously injected of Se6‐NPs/Ce6‐NPs on tail every 2 days, while white light irradiation every 48 h as well. Concentrations of Se6‐NPs/Ce6‐NPs: 100 µm in 100 µL. b) Changes of Mice weight with various treatments during the PDT study period. c) Changes of tumor volume with various treatments during the PDT study period of 21 days, **p < 0.01. d) Photos of tumor in vitro at day 21 after various treatments. e) Statistics of in vitro tumor weight at day 21 after various treatments, ****p < 0.0001, ***p < 0.001, and *p < 0.05. f) Images of HE, TUNEL, and Ki67 staining analysis of tumor slices. Scale bars: 100 µm; various treatments (*n* = 4, mean ± SD): PBS for Group 1, PBS + Light (L) for Group 2, PBS + Se6‐NPs for Group 3, PBS + Se6‐NPs + L for Group 4 and PBS + Ce6‐NPs + L for Group 5.

The in vivo phototherapeutic study of Se6‐NPs on 4T1 cells was conducted continuously. In consideration of long‐term accumulation in tumors of Se6‐NPs, Se6‐NPs were intravenously injected on thetail every 2 days (48 h), while tumor sites were exposed to a dose of white light irradiation in 24 h after every injection, as illustrated in **Figure** **7**a. Such treatment could help to minimize the suffering of injection treatments. Thus the mice's weights and the tumor volumes were tracked every 2 days, to evaluate the inhibiting effect on tumor growth (Figure 7b,c). As shown in Figure 7c, the curves indicated that aggressive growth of tumors in control groups was observed during the study period. And no significant difference in tumors growth was shown among these groups, including phosphate buffer saline (PBS) (Group 1), PBS + light (L) (Group 2), and PBS + Se6‐NPs (Group 3). Surprisingly, both Se6‐ and Ce6‐NPs would exhibit obvious TGI, upon the introduction of white light irradiation. Especially for Se6 in group of PBS + Se6‐NPs + L (Group 4), there was almost no tumor growth observed, demonstrating better PDT performance of Se6 than the commercial PS of Ce6. As shown in Figure 7e and Figure S22a, Supporting Information, the weight of dissected tumors at the end of 21 days oftreatments in Group 4 was much smaller than these in both three control groups and Group 5 (PBS + Ce6‐NPs + L), further confirming the excellent TGI efficiency of Se6‐NPs. Actually, almost no tumors could be found in half of mice in Group 4 with Se6‐NPs + L, as shown in Figure 7d. And the calculated TGI rates of 99% for Se6‐NPs were also higher than that of 79% for Ce6‐NPs, further confirming that the newly designed type‐I PS of Se6 possessed highly efficient capability on significantly inhibiting tumor growth upon irradiation.

In order to deeply evaluate the PDT performance on Se6, immunohistochemistry of Se6 and Ce6 had been carried out (Figure 7f and Figure S22b, Supporting Information). As shown in the hematoxylin and eosin (HE) staining results of the dissected tumors, destruction of tumor tissue was obviously observed in Group 4 with Se6‐NPs + L and Group 5 with Ce6‐NPs + L. And large‐area cell apoptosis of tumor tissue induced by Se6‐NPs + L or Ce6‐NPs + L was further verified by the plenty of green fluorescence signals in terminal‐deoxynucleotidyl transferase‐mediated nick end labelling (TUNEL) straining assay on tumor tissue section (Figure 7f and Figure S22b, Supporting Information). The monoclonal antibody Ki67 staining assay was also performed and few Ki67‐positive proliferating cells were found in Group 4 and Group 5, supporting the PDT results observed in HE and TUNEL staining as well. These results convincingly demonstrated that Se6 possessed ahigh efficient capability on inhibiting tumor growth by inducing tumor cells apoptosis and subsequently restraining their proliferation upon irradiation. In addition, there were no abnormal body weight changes in treated mice in each group during the period of thePDT course, indicating small effects on the health of treated mice (Figure 7b). At the end of in vivo PDT treatments, the histological analysis of themain organs of the mice was also performed and no any noticeable inflammatory damage in various organs was observed, suggesting appreciable biocompatibility and biosecurity of Se6‐NPs (Figure S23, Supporting Information).

## 3 Conclusion

In conclusion, two excellent selenium‐containing type‐I PSs of Se6 and Se5 have been designed, which were found to exhibit highly efficient generation of dual type‐I radicals upon irradiation and AIE properties. The Se‐containing BSeD acceptor has been introduced into two PSs, which was proved to bring them both strong effects of ICT and heavy atom. Benefiting from double heavy‐atom‐effect of Se and Br, Se6 possessed asmaller Δ*E*_ST_ of 0.03 eV, leading to ahigher population of excited triplet PS and subsequent improved ROS efficiency. Interestingly, type‐I radicals of both O_2_^•‾^ and ^•^OH but no type‐II radical of ^1^O_2_ could be highly efficient generation from Se6 and Se5 upon irradiation with white light, which might provide them witha switch‐hitter of dual‐radical with complementary lifetimes for outstanding PDT in hypoxia condition. Importantly, two simultaneous processes to produce ^•^OH radicals with or without oxygen participation were revealed, including superoxide disproportionation of O_2_^•‾^ and reaction between excited PS and water. Therefore, Se6 and Se5 actually could produce ^•^OH efficiently without the help of any oxygen, obtaining a complete remission of oxygen for PDT. In vitro experiments demonstrated that these NPs of Se6 or Se5 displayed comparable high photocytotoxicity under hypoxia condition to these of normoxia condition, conforming totheir small dependence on oxygen. Notably, Se6‐NPs with double heavy‐atom effect of Se and Br exhibited superior PDT performance, even better than the commercial Ce6‐NPs, either normoxia or hypoxia conditions. In addition, Se6‐NPs simultaneously achieved long‐term accumulation in tumor tissue and appreciable biosecurity in the mouse model. Therefore, after intravenously injection, a significant PDT performance and anticancer outcome was demonstrated on Se6‐NPs, with higher TGI rates of 99% than the commercial Ce6‐NPs as well. The findings in this study would offer new insights into both the molecular design and mechanism study of type‐I PSs, which trigger developments of advanced hypoxia PDT in future cancer treatments.

## Conflict of Interest

The authors declare no conflict of interest.

## Supporting information

Supporting Information

## Data Availability

The data that support the findings of this study are available in the supplementary material of this article.
