# Supplementary material for: Selenium‐Containing Type‐I Organic Photosensitizers with Dual Reactive Oxygen Species of Superoxide and Hydroxyl Radicals as Switch‐Hitter for Photodynamic Therapy
Source: Adv Sci (Weinh). 2023 Jun 25;10(24):2301902. doi: 10.1002/advs.202301902 (PMC10460872; doi:10.1002/advs.202301902)
Supplement: Supplementary file 1 — Supporting Information [file ADVS-10-2301902-s001.pdf]

ADVANCED SCIENCE
Open Access

Supporting Information

for *Adv. Sci.*, DOI 10.1002/advs.202301902

Selenium-Containing Type-I Organic Photosensitizers with Dual Reactive Oxygen Species of Superoxide and Hydroxyl Radicals as Switch-Hitter for Photodynamic Therapy

Haiyang Wang, Tian Qin, Wen Wang, Xie Zhou, Faxu Lin, Guodong Liang, Zhiyong Yang, Zhenguo Chi and Ben Zhong Tang*

Supplementary Information

Selenium-containing Type-I Organic Photosensitizers with Dual Reactive Oxygen Species of Superoxide and Hydroxyl Radicals as Switch-Hitter for Photodynamic Therapy

Haiyang Wang[+], Tian Qin[+], Wen Wang, Xie Zhou, Faxu Lin, Guodong Liang, Zhiyong Yang[*], Zhenguo Chi and Ben Zhong Tang

Experimental materials and methods：

1.1 General materials

***Materials**:* Distearoyl phosphoethanolamine-PEG2000 (DSPE-PEG2000) was purchased from Xi'an ruixi Biological Technology Co., Ltd. Methylene blue (MB), 5,5-dimethyl-1-pyrroline-*N*-oxide (DMPO) and 9,10-anthracenediylbis(methylene) dimalonic acid (ABDA) were obtained from Shanghai Aladdin Biochemical Technology Co., Ltd. Fetal bovine serum (FBS) and basal medium were purchased from Thermo Fisher Scientific Co., Ltd. 2',7'-Dichlorodihydrofluorescein diacetate (DCFH-DA) was got from Dalian Meilun Biotechnology Co., Ltd. Reactive oxygen species (ROS) fluorescence sensor of Brite™ HPF (HPF) was purchased from AAT Bioquest. Dihydrorhodamine 123 (DHR123), ROS fluorescent probe-DHE (DHE), lysosome probe (Lyso-Tracker Green), endoplasmic reticulum probe (ER-Tracker Green), and mitochondria probe (Mito-Tracker Green), JC-1 mitochondrial membrane potential probe, and Annexin V-FITC/PI (fluorescein isothiocyanate/propidium iodide) double staining apoptosis detection kit were purchased from Jiangsu Kaiji Biotechnology Co., Ltd. Cell Counting Kit-8 (CCK8) was obtained from Dongren Chemical Technology Co., Ltd. Chlorin e6 (Ce6) was purchased from Frontier Scientific. Unless otherwise noted, molecular synthesis reagents were purchased from bidepharm, Adamas (Tansoole platform), Energy Chemical, J & K Chemical, or Aladdin Chemical and used without further purification.

Characterizations and instruments: All reactions were performed under an inertia atmosphere unless otherwise specified. Flash chromatography was performed using silica gel (300-400 mesh). Analytical thin-layer chromatography was performed on

0.20 mm silica gel HSGF-254 plates (Huanghai, China) and visualized under 254 or 365 nm UV light. Column chromatography was performed using 200-300 mesh silica gel (Huanghai, China). ^{1}H and ^{13}C nuclear magnetic resonance (NMR) spectra were obtained at 25 °C on a Bruker Advance III 400 MHz NMR spectrometer. Chemical shifts were reported in parts per million (ppm) with reference to TMS or residual nuclei in deuterated solvents [^{1}H NMR ($CDCl_3$): δ =7.26 ppm; ^{13}C NMR ($CDCl_3$): δ = 77.2 ppm]. Coupling constants (*J*) are denoted in Hz. Multiplicities are denoted as follows: s = singlet, d = doublet, dd = doublet of doublets, t = triplet, q = quartet, m = multiplet, br = broad. MALDI-TOF mass spectra were recorded on an UltrafleXtreme MALDI-TOF/TOF instrument (Bruker Daltonics, Germany) equipped with a 355 nm Nd: YAG laser. The particle size distribution and its polydispersity index (PDI) were obtained from the dynamic light scattering (DLS) detection based on a Malvern nano ZS Panalytical Zetasizer. Photoluminescence (PL) spectra were measured using a Shimadzu RF-5301PC spectrometer or a Horiba fluoromax4 spectrometer. UV-vis absorption spectra were detected using a Hitachi U-3900 spectrophotometer. Electron ParaMagnetic Resonance (EPR) signals were collected from a Bruker A300 Electron ParaMagnetic Resonance. Scanning electron microscope (SEM) imaging of nanoparticles were observed on a Hitachi S-4800 scanning electron microscope. Confocal imagings of cells were observed on an Olympus FV3000 confocal microscope. Flow cytometry analysis was performed using an Attune Nxt flow cytometer. Nanoparticle sizes were measured on a Malvern Panalytical Zetasizer nano. CCK8 UV absorptions were collected from a Synergy H1 multifunctional microplate reader. In vivo imagings were collected from PerkinElmer IVIS Lumina III Series. The quantum chemistry calculationd were performed on the Gaussian 09 software at B3LYP/6-31G(d) level of theory using the density functional theory (DFT) method. The energy levels of excited states were caculated from the corresponding ground state geometry using the combination of TD-B3LYP/6-31G(d).

2 Synthesized and Measured Methods

2.1.1 Synthesis of Se6, Se5 and SeH：

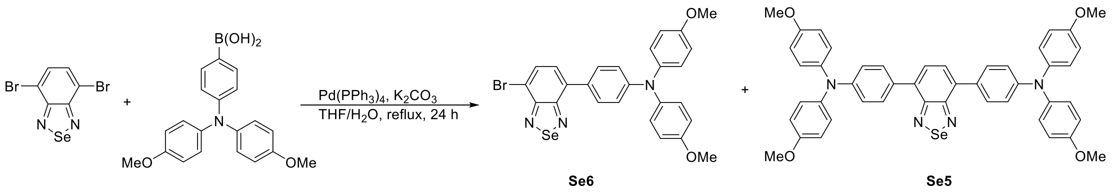

Scheme S1. Synthetic routes of Se6 and Se5.

The compound of S5 was prepared based on the known procedure.[1]

4,7-Dibromo-2,1,3-benzoselenadiazole were prepared according to the literature report.[2] Added 4,7-dibromo-2,1,3-benzoselenadiazole (0.34 g, 1.0 mmol), [4-[bis(4-methoxyphenyl)amino]phenyl]boronic acid (0.45 g, 1.3 mmol), $Pd(PPh_3)_4$ (0.24 g, 0.2 mmol), and anhydrous potassium carbonate (1.1 g, 8.0 mmol) into tetrahydrofuran water mixed solvent [30 ml (v/v = 4: 1)] and then refluxed 24 h under nitrogen condition. Subsequently, the reaction was extracted with dichlromethylane (DCM) for three times and then washed with brine. The organic layer was dried with anhydrous sodium sulfate. The filtrate was then evaporated under vacuum and purified by column chromatography [petroleum ether (PE) : DCM = 6:1 to 1:1] to give Se6 (0.31 g, 53 %) and Se5 (0.13 g, 16 %) as red soilds. ^{1}H NMR (Se6, 400 MHz, $CDCl_3$) δ = 7.80 (d, *J* = 7.5 Hz, 1H), 7.66 (d, *J* = 8.4 Hz, 2H), 7.34 (d, *J* = 7.4 Hz, 1H), 7.13 (d, *J* = 8.1 Hz, 4H), 7.01 (d, *J* = 8.1 Hz, 2H), 6.86 (d, *J* = 8.7 Hz, 4H), 3.81 (s, 6H). ^{13}C NMR (101 MHz, $CDCl_3$) δ = 158.7, 156.4, 140.5, 135.6, 132.6, 130.1, 127.3, 119.5, 114.9, 114.6, 55.6. MALDI-TOF-MS m/z calcd. for $C_{26}H_{20}BrN_3O_2Se$ [M]: 564.99, found: 564.99.

^{1}H NMR (Se5, 400 MHz, $CDCl_3$) δ = 7.72 (d, *J* = 8.6 Hz, 4H), 7.53 (s, 2H), 7.13 (d, *J* = 8.6 Hz, 9H), 7.04 (d, *J* = 8.4 Hz, 4H), 6.86 (d, *J* = 8.8 Hz, 8H), 3.81 (s, 12H). ^{13}C NMR (101 MHz, $CDCl_3$) δ = 160.1, 156.2, 148.8, 140.8, 134.0, 127.7, 127.1, 119.9, 114.9, 55.6. MALDI-TOF-MS m/z calcd. for $C_{46}H_{38}N_4O_4Se$ [M]: 790.21, found: 790.21.

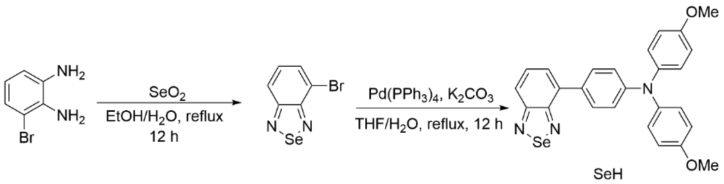

Scheme S2. Synthetic routes of SeH.

4-Bromobenzo[c][1,2,5]selenadiazole was prepared according to the literature report.[1] 4-bromobenzo[c][1,2,5]selenadiazole (0.26 g, 1.0 mmol), [4-[bis(4-methoxyphenyl)amino]phenyl]boronic acid (0.45 g, 1.3 mmol), $Pd(PPh_3)_4$ (0.24 g, 0.2 mmol), and anhydrous potassium carbonate (1.1 g, 8.0 mmol) into tetrahydrofuran water mixed solvent [30 ml (v/v = 4: 1)] and then refluxed 12 h under nitrogen condition. Subsequently, the reaction was extracted with DCM for three times and then washed with brine. The organic layer was dried with anhydrous sodium sulfate. The filtrate was then evaporated under vacuum and purified by column chromatography [petroleum ether (PE) : DCM = 6:1 to 1:1] to give SeH (0.35 g, 73 %) as orange-red soilds. ^{1}H NMR (SeH, 400 MHz, $CDCl_3$) δ = 7.77 (d, *J*=8.7, 1H), 7.71 (d, *J*=8.6, 2H), 7.53 (dd, *J*=8.8, 6.9, 1H), 7.46 (d, *J*=6.4, 1H), 7.14 (d, *J*=8.6, 4H), 7.04 (d, *J*=8.4, 2H), 6.87 (d, *J*=8.9, 4H), 3.82 (s, 6H). ^{13}C NMR (101 MHz, $CDCl_3$) δ = 156.26, 149.01, 140.73, 135.98, 130.17, 129.55, 127.15, 126.98, 121.81, 119.75, 114.89, 55.64. MALDI-TOF-MS m/z calcd. for $C_{26}H_{21}N_3O_2Se$ [M]: 487.08, found: 487.07.

2.1.2 Preparation of Se6-NPs and Se5-NPs: 1 mg Se6 or Se5 and 3 mg DSPE-PEG2000 were dissolve in 1.0 ml THF according to the weight ratio of 1:3. Slowly added the THF solution into 10 ml water under ultrasonic conditions. After the dripping was completed, the mixture was sonicated for 5 min. The excess THF was removed by dialysis using a dialysis bag (MWCO: 10, 000 Da) for 48 h in water.

2.2.1 The overall ROS detection by DCFH: The overall ROS generation measurement was conducted using DCFH as a fluorescence sensor. Se6-NPs/Se5-NPs (30 μM) and DCFH (25 μM) were dispersed in PBS solution. White light (20 mW/cm^2) was used to

irradiate the solution, and the change in fluorescence of these solutions were detected by a fluorescence spectrophotometer at an interval of 20 s.

2.2.2 The singlet oxygen ($^1O_2$) detection by ABDA: The singlet oxygen generation measurement was conducted using ABDA as a sensor. MB, Se6-NPs/Se5-NPs (30 μM) and ABDA (100 μM) were dispersed in PBS solution. White light (20 mW/cm^2) was used to irradiate the solution, and the change on UV absorption of ABDA of these solutions were measured on a UV spectrophotometer at an interval of 20 s. Especially for Ce6, Ce6 (30 μM) and ABDA (100 μM) were dispersed in different solution (H_2O, dimethyl sulfoxide (DMSO), H_2O/DMSO (1/1, V/V)).

2.2.3 The superoxide anion ($O_2^{•-}$) detection by DHR123: The superoxide anion generation measurement was conducted using DHR123 as a fluorescence sensor. Se6-NPs/Se5-NPs (30 μM) and DHR123 (10 μM) were dispersed in PBS solution. White light (20 mW/cm^2) was used to irradiate the solution, and the change in fluorescence of these solutions were detected by a fluorescence spectrophotometer at an interval of 20 s.

2.2.4 The hydroxyl radical ($^{•}OH$) detection by HPF: The hydroxyl radical generation measurement was conducted using HPF as a fluorescence sensor. Se6-NPs/Se5-NPs (30 μM) and HPF (20 μM) were dispersed in PBS solution. White light (20 mW/cm^2) was used to irradiate the solution, and the change in fluorescence of these solutions were detected by a fluorescence spectrophotometer at an interval of 1 min.

For hypoxic condition, the mixture solution of HPF with Se6-NPs/Se5-NPs was deoxygenized through three cycles of a sequential procedure involving cryofreezing, vacuuming, and defrosting. Then, high purity of nitrogen gas was used to aerate the sample. The irradiation by white light and subsequent measurement was carried out under the nitrogen atmosphere.

2.2.5 The superoxide anion ($O_2^{•-}$) detection by Electron ParaMagnetic Resonance (EPR): The generation of $O_2^{•-}$ was qualitatively characterized using an EPR spectrometer. For the hypoxic condition, Se6/Se5 (100 μM) and DMPO were dissolved in DMSO. The mixed solution was exposed to white light (0.1 W/cm^2) for 5 min. The

product of DMPO-$O_2^{\bullet-}$ was immediately tracked with EPR spectrometer.

For the normoxia condition, Se6/Se5 (100 μM) and DMPO were also dissolved in DMSO. When exposed to white light (0.1 W/cm^2) for 5 min, air was continuously bubbling into the mixed solution at the same tiem, in order to increase the oxygen content of the solution. The product of DMPO-$O_2^{\bullet-}$ was immediately tracked with EPR spectrometer.

2.2.6 The hydroxyl radical (•OH) detection by Electron ParaMagnetic Resonance (EPR): For the normoxia condition, Se6-NPs/Se5-NPs (100 μM) and DMPO were dissolved in PBS solution. The mixed solution was exposed to white light (0.1 W/cm^2) for 5 min. The product of DMPO-•OH was immediately tracked with EPR spectrometer.

For the hypoxic condition, Se6-NPs/Se5-NPs (100 μM) and DMPO were also dissolved in PBS solution. the mixture solution was deoxygenized through three cycles of a sequential procedure involving cryofreezing, vacuuming, and defrosting. High purity of nitrogen gas was subsequently used to aerate the sample. Then, the mixed solution was exposed to white light (0.1 W/cm^2) for 5 min. The product of DMPO-•OH was immediately tracked with EPR spectrometer.

2.3 Cell culture ***:*** The 4T1 (or RM-1) cells were cultured in 1640 medium containing 10% FBS and antibiotics (100 units/mL penicillin and 100 μg/mL streptomycin) in a humidified incubator with 5% CO_2 at 37 ℃.

2.3.1 Cell localization experiment: The 4T1 cells were seeded in a confocal dish. The nanoparticles and the cells were incubated for different times, stained with lysosome probe (LysoGreen), and then fixed with paraformaldehyde. The position distribution of nanoparticles in the cell were observed by using a laser confocal microscope.

2.3.2 Cytotoxicity test: The 4T1 cells were inoculated in a 96-well plate (1×10^4 per well). After the cells adhered to the wall, the cells were co-cultured with different concentrations of Se6-NPs, Se5-NPs and Ce6-NPs for 4 h, respectively. Then they were irradiated with white light (20 mW/cm^2) for 30 min, and were continued culturing for 24 h. The CCK8 was utilized to detect the cell viability of these samples. For the

hypoxic cytotoxicity test, the adherent cells were put in a hypoxic incubator for 6 h. Then different concentrations of Se6-NPs, Se5-NPs and Ce6-NPs were added into and co-cultured with the cells for 4 h, respectively. These samples were irradiated with white light (20 mW/cm^2) for 30 min, and continued culturing for 24 h. The cell viability was also detected by CCK8. Regarded to the RM-1 cells, similar procedures to 4T1 cells were performed with different concentrations of Se6-NPs and Se5-NPs. All nanoparticles were performed six samples at the same time.

2.3.3 Cell apoptosis analysis: The 4T1 cells were inoculated in the culture plate. After the cells adhered to the wall, the cells were co-cultured with Se6-NPs or Se5-NPs for 4 h. Then they were irradiated with white light (20 mW/cm^2) for 30 min, and continued culturing for 24 h. The apoptosis kit (Annexin V-FITC/PI) was utilized to stain and the cell apoptosis was observed by using a flow cytometry or a confocal laser scanning microscope (CLSM).

2.3.4 Intracellular ROS detection: The cells were inoculated in a confocal culture dish. After the cells adhered to the wall, the cells were co-cultured with Se6-NPs for 4 h. DCFH-DA, HPF, and DHE fluorescence probes were co-cultured with the cells for 30 min, and then these cells were irradiated with white light (20 mW/cm^2) for 10 min. The ROS production in the cells was observed by a confocal microscope. The cells in corresponding hypoxia group were under the hypoxia condition for 6 h in advance. The cells were also kept in a hypoxia environment when they were irradiated, and then the observations of cells were performed subsequently on a confocal microscopy.

2.3.5 Mitochondrial membrane potential (MMP) measurement: The MMP of cells was detected by JC-1 mitochondrial membrane potential probe in accordance with the manufacturer’s protocol. Briefly, after different treatments, cells were incubated with 2 μg/mL of JC-1 probe for 20 min at 37 °C. After washing twice with PBS, the cells were observed using a confocal laser scanning microscope (CLSM).

2.3.6 Confocal co-localization: The 4T1 cells were inoculated in a confocal culture dish. After the cells adhered to the wall, the cells were co-cultured with Se6-NPs or Se5-NPs for 4 h. and Then cells were incubated with 1 mL medium containing commercial dyes at 37 °C for 30 min, including Lyso-Tracker Green (100 nM, stock

solution: 1 mM in DMSO), ER-Tracker Green (1 μM, stock solution: 1 mM in DMSO) or Mito-Tracker Red (100 nM, stock solution: 100 μM in DMSO), respectively. The medium was then removed. After rinsed with PBS for three times, the cells were observed using CLSM. For Se6-NPs and Se5-NPs, the excitation was 488 nm and the emission filter was 650–690 nm; For probes, ER-Tracker Green, Mito-Tracker green and Lyso-Tracker green, the excitation was 488 nm, and the emission filter was 510–540 nm.

2.4 Experimental animal models: The animal experimental program was approved by the animal ethics committee of Sun Yat-sen University. The female BALB/c mice (6 weeks old) were purchased from Guangdong medical laboratory animal center. 4T1 cells (5×10^5) suspended in PBS solution were subcutaneously injected into the mouse. The tumor treatment was started when the tumor volume was close to 70 mm^3 after 7 days of inoculation.

2.4.1 In-vivo cancer treatment assessment: The tumor-bearing mice were randomly divided into five groups (four mice in each group, n = 4): PBS, PBS + Light, Se6-NPs, Se6-NPs + Light and Ce6-NPs + Light. They were intravenously injected with nano-drug (100 μM, 100 μL) or PBS (100 μL) on tail every two days (48 h). The body weight and the tumor volume were recorded every two days during the experimental period. After 21 days, the tumor-bearing mice were killed and their tumor tissues and viscera were examined by immunohistochemical staining.

2.4.2 In-vivo fluorescent assessment: In brief, the tumor-bearing mice were injected with 100 μL Se6-NPs (Se6 100 μM) in caudal vein, and then observed the distribution of *in-vivo* fluorescence at different time points using an In Vivo Imaging System (IVIS). After the mice dissected 48 h later, its major organs were removed to observe fluorescence distribution. Wavelengths for excitation and emission: 520 and 670 nm.

2.4.3 Pharmacokinetic study: To evaluate the pharmacokinetics of Se6-NPs, three healthy female BALB/c mice were intravenously injected with Se6-NPs (100 μL, 100 μM). Then their blood were collected at different time points and the relative blood serum was provided by a centrifuge. Then 10 μL of blood serum was taken from each

sample for the assay. The fluorescence of blood serum fluid was examined using a multifunctional microplate reader. After 48 h injection of Se6-NPs, the distribution of Se6-NPs in major organs was then observed using an In Vivo Imaging System (IVIS). Wavelengths for excitation and emission: 520 and 670 nm.

3 Figures and Tables

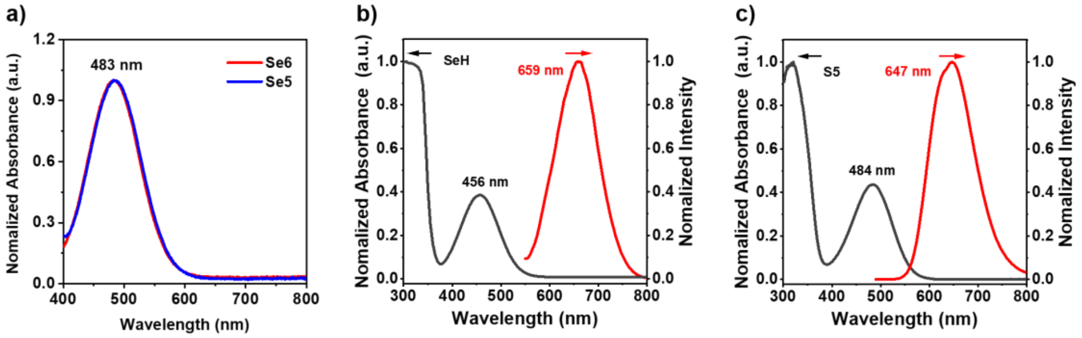

Figure S1. a) The UV-vis absorption spectra of Se5 and Se6 in THF solution (10 μM). The UV-vis absorption and PL spectra of b) SeH and c) S5 in THF solution (10 μM).

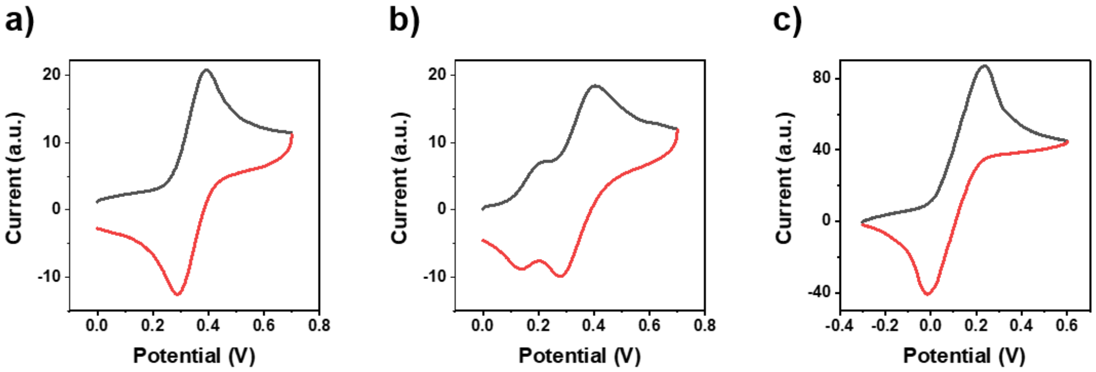

Figure S2. The cyclic voltammograms of a) Se6, b) Se5 and c) Ferrocene. Concentrations: 1 mM.

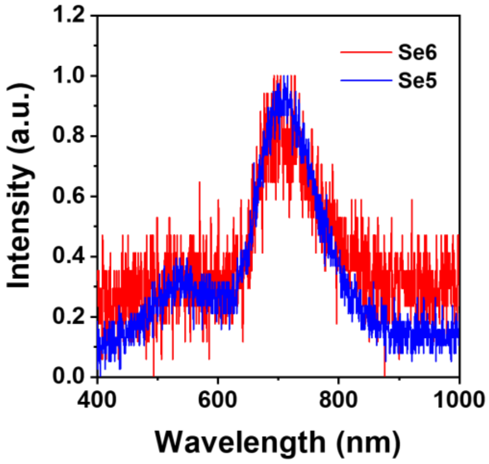

Figure S3. The phosphorescence spectra of Se5 and Se6 in solid state at 77 K.

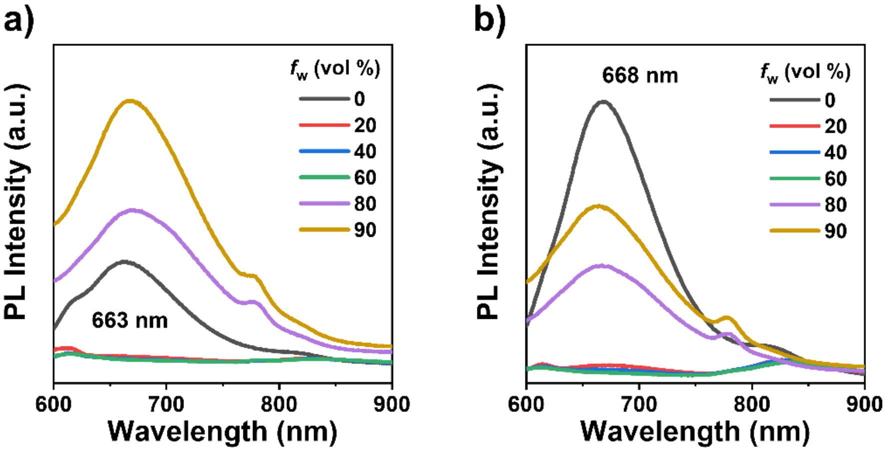

Figure S4. The PL spectra of a) Se6 and b) Se5 (10 μM) in THF/water mixtures with different water fractions (f_w).

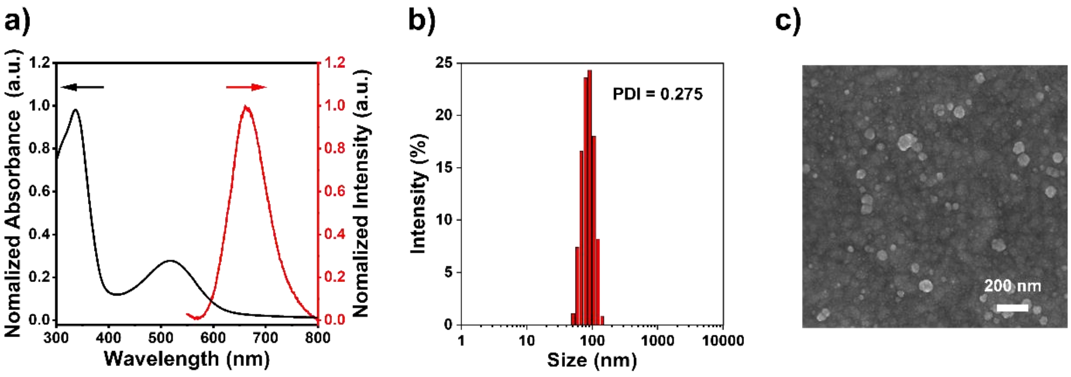

Figure S5. a) UV and PL spectra (10 μM), b) Particle size distribution, and c) SEM image of Se5-NPs. Scale bar in SEM image: 200 nm. The PDI of nanoparticles was also inset in b).

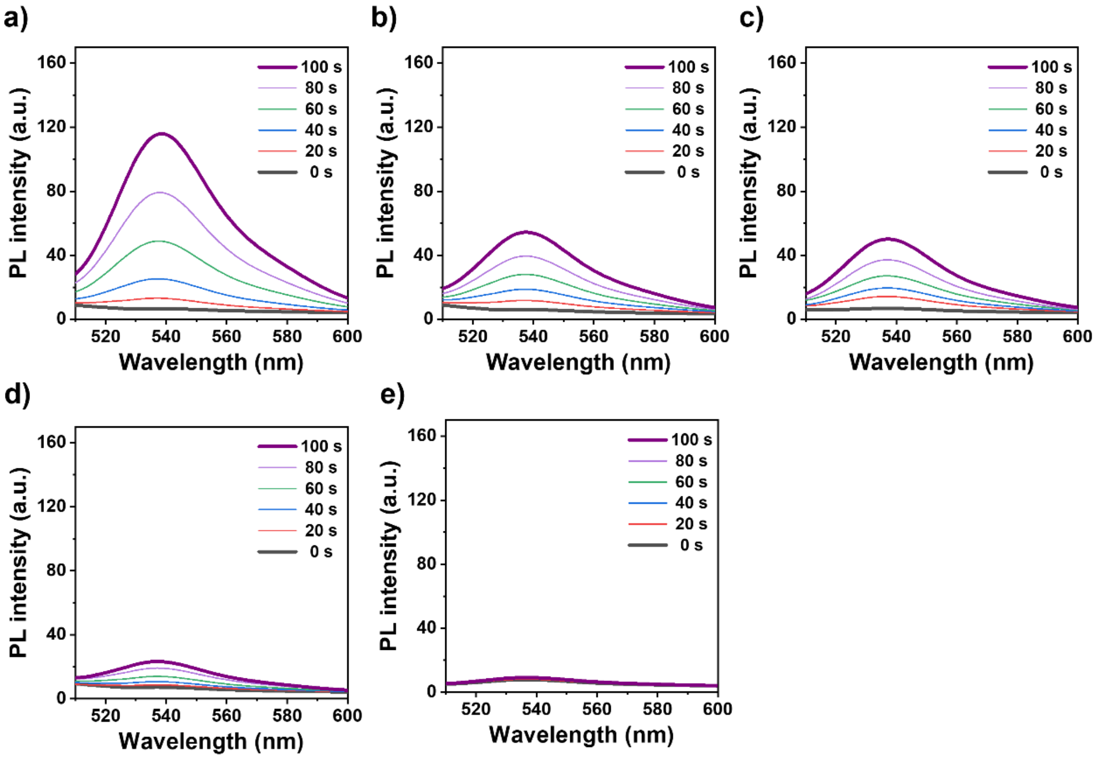

Figure S6. PL spectra of DCFH in PBS in the present of a) Se6, b) Se5, c) SeH, d) S5, and e) Blank (DCFH alone) after exposure to white light irradiation of 20 mW cm^{-2} with different time. Concentrations: 30 μM for Se6, Se5, SeH and S5; 25 μM for DCFH.

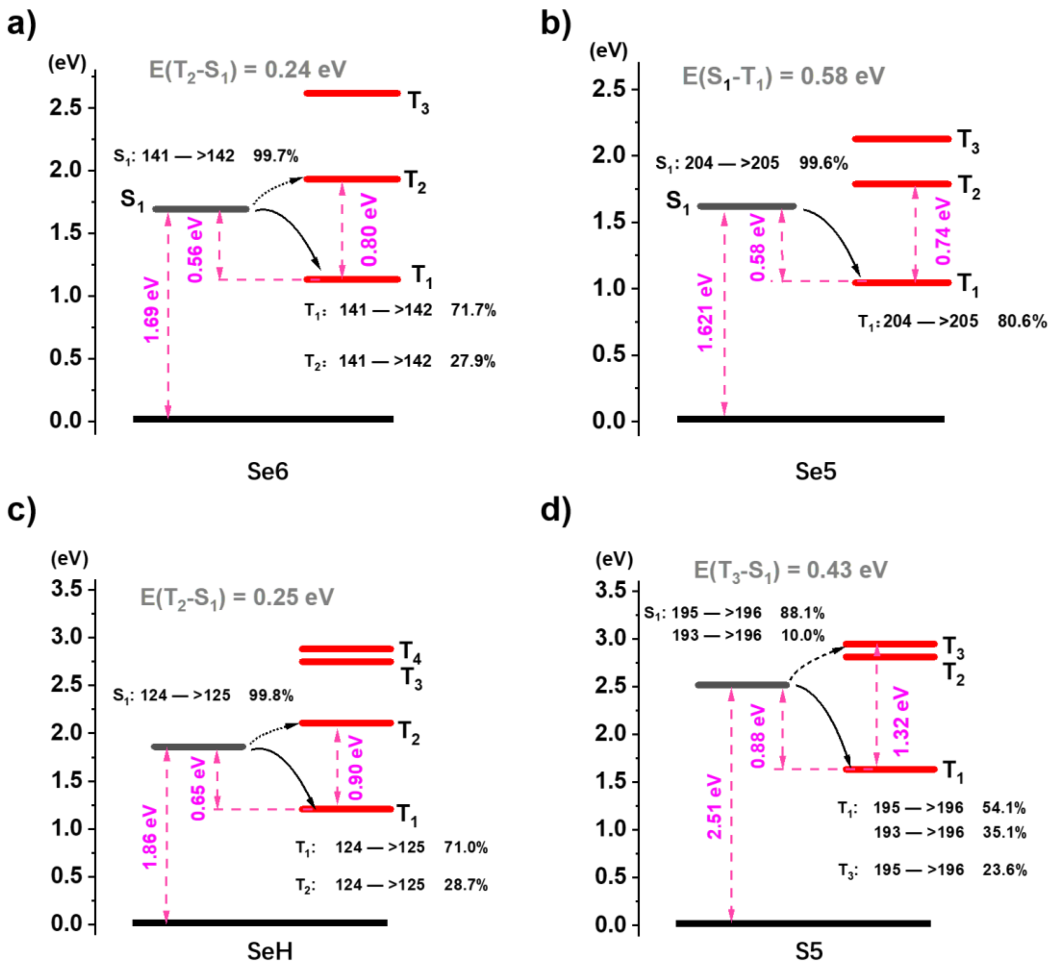

Figure S7. DFT calculated energy levels arrangement and molecular orbitals transitions inside both S_1 and T_1 of a) Se6, b) Se5, c) SeH, and d) S5.

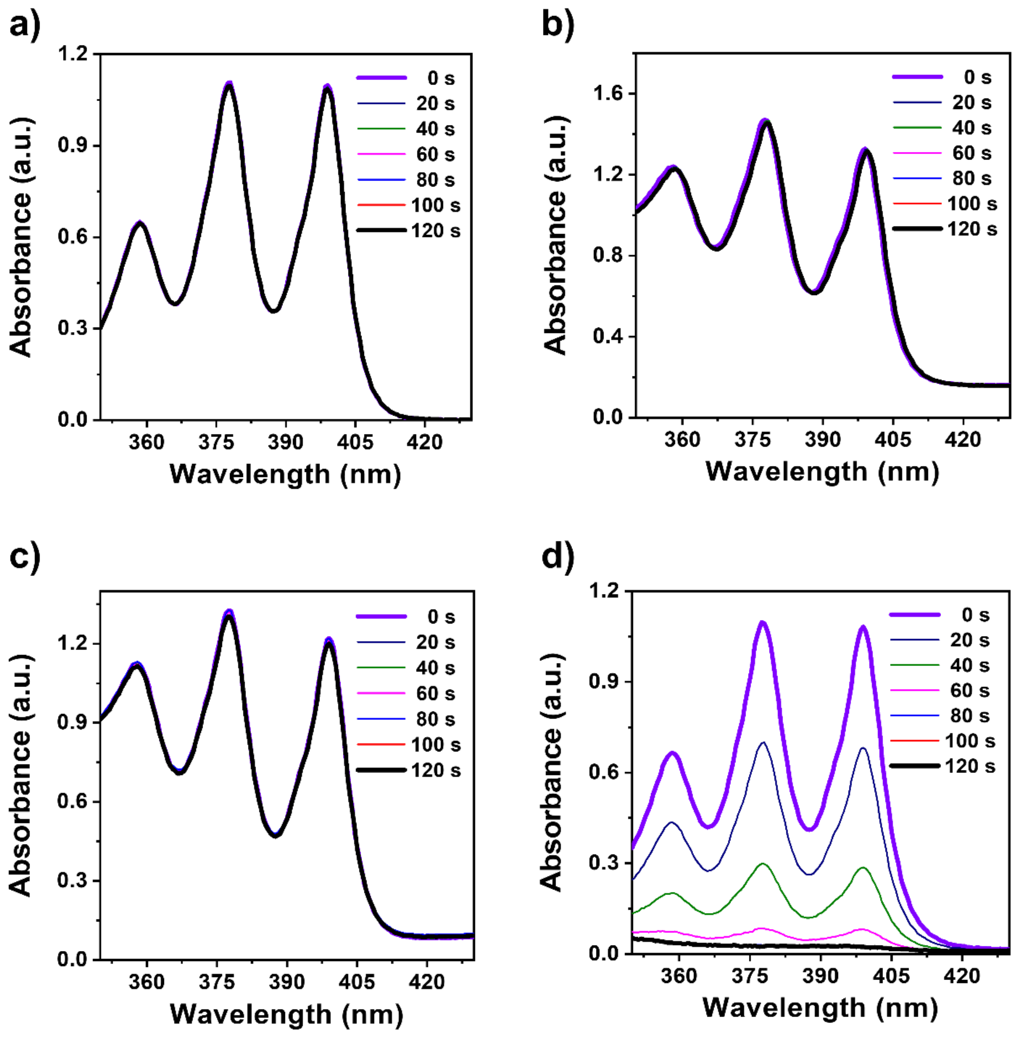

Figure S8. Absorption spectra of ABDA in PBS in the presence of a) Se6-NPs, b) Se5-NPs, c) ABDA alone, and d) MB after exposure to white light irradiation of 20 mW cm^{-2} with different time. Concentrations: 30 μM for MB, Se5-NPs and Se6-NPs, 100 μM for ABDA.

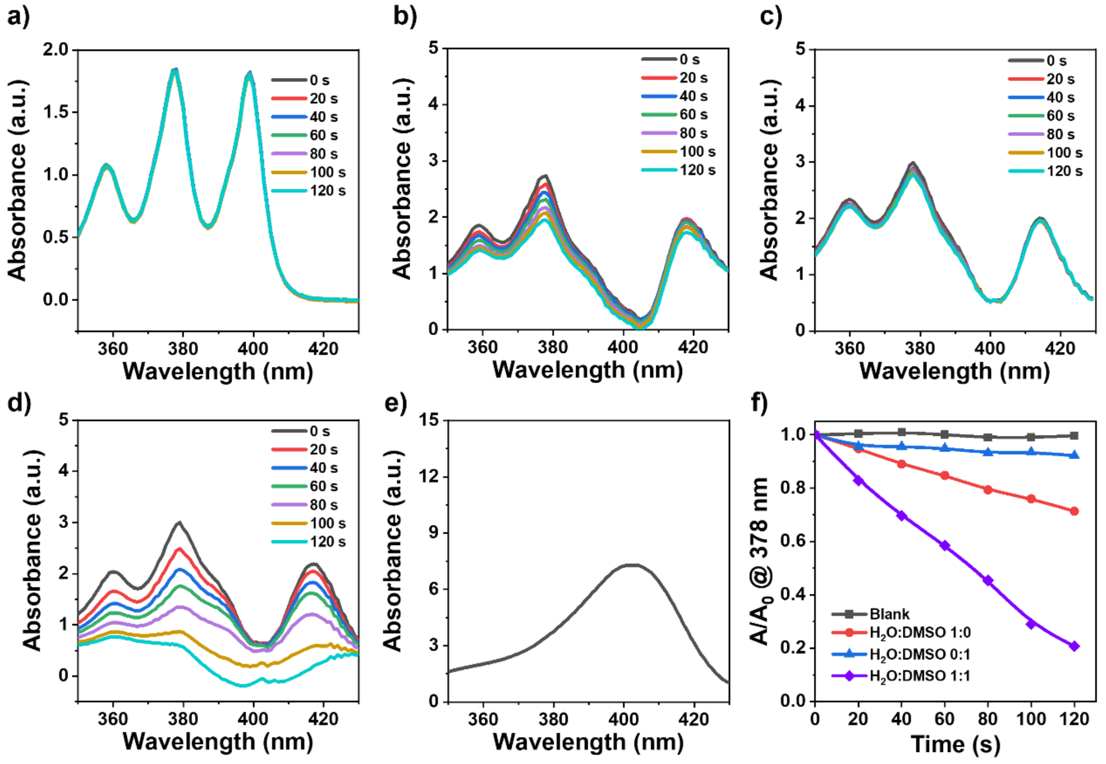

Figure S9. The singlet oxygen generation detection of Ce6 in various solvents. Absorption spectra of ABDA in a) water, and in the presence of Ce6 in b) water, c) DMSO, and d) water/DMSO = 1/1 (V/V) after exposure to white light irradiation of 20 mW cm^2 with different time. e) UV absorption of Ce6 in DMSO. f) The plots of decomposition rates of ABDA in solution with various ratio of water and DMSO. Concentration: 30 μM for Ce6, 100 μM for ABDA.

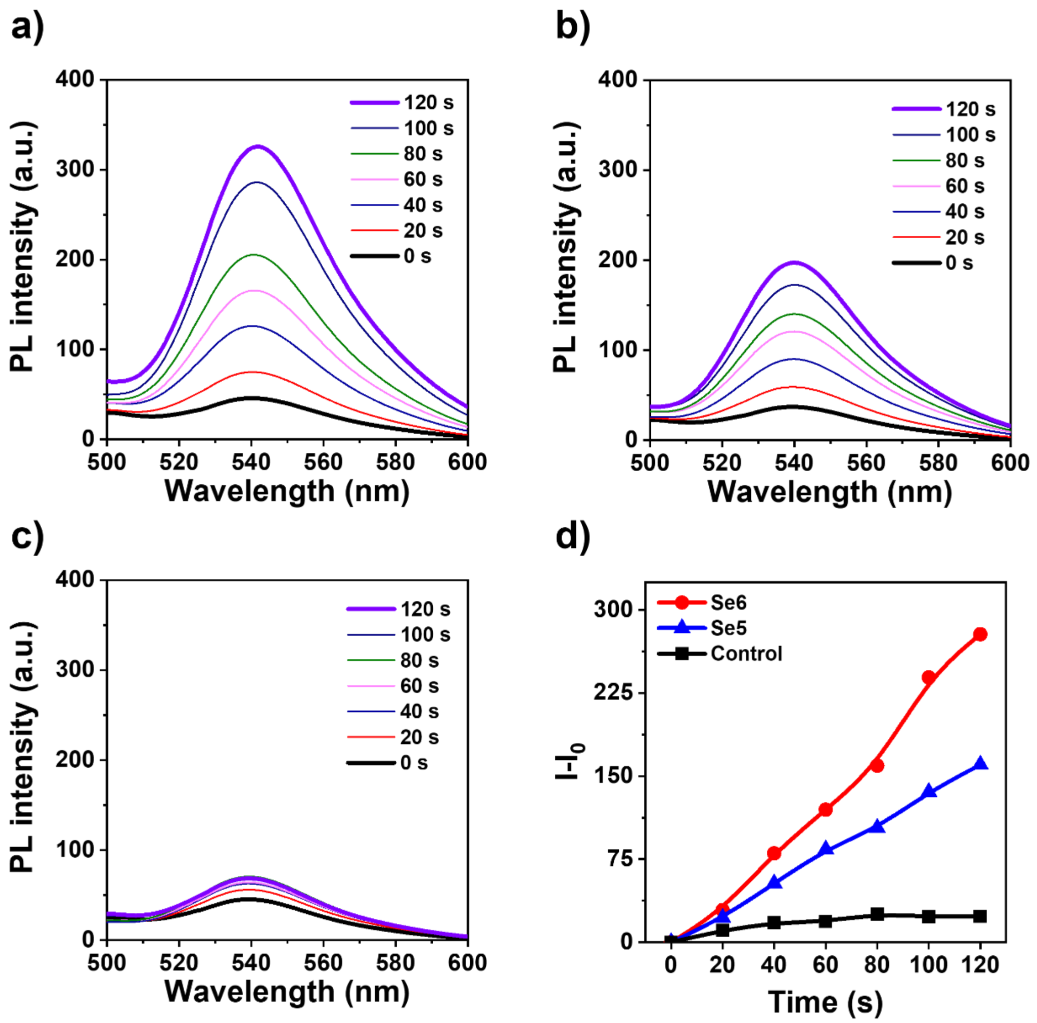

Figure S10. PL spectra of DHR123 in PBS in the present of a) Se6-NPs, b) Se5-NPs, and c) DHR123 alone after exposure to white light irradiation of 20 mW cm^{-2} with different time. d) Plot of relative PL intensity of $O_2^{\bullet -}$ probe DHR123 versus irradiation time. Concentrations: 30 μM for Se5-NPs and Se6-NPs, 10 μM for DHR123.

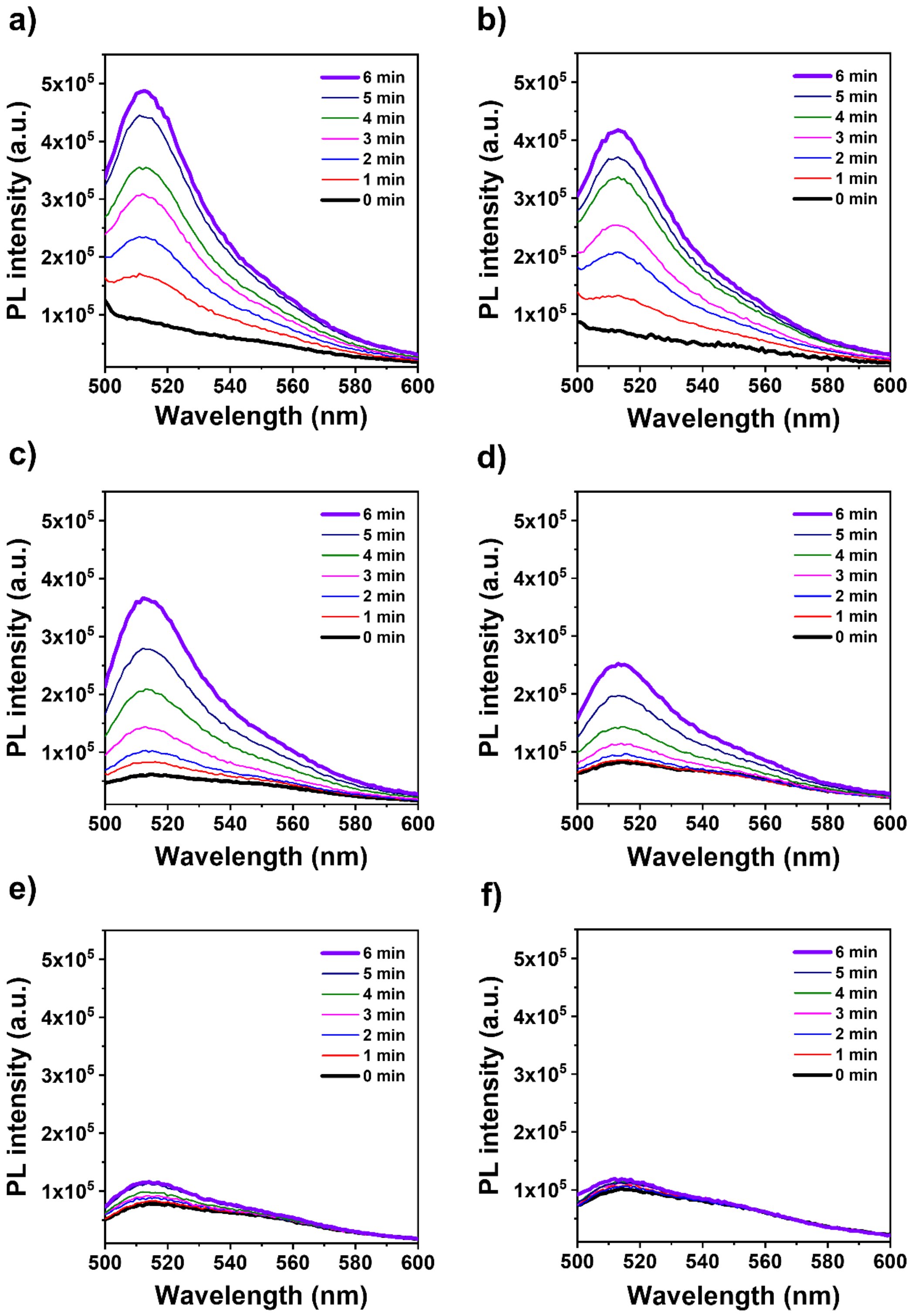

Figure S11. PL spectra of HPF in PBS in the presence of PSs under the white light irradiation of 20 mW cm^{-2} with different time: a) Se6-NPs, c) Se5-NPs, and e) HPF alone under normoxia condition; b) Se6-NPs, d) Se5-NPs, and f) HPF alone under hypoxia condition. Concentrations: 30 μM for Se5-NPs and Se6-NPs, 20 μM for HPF.

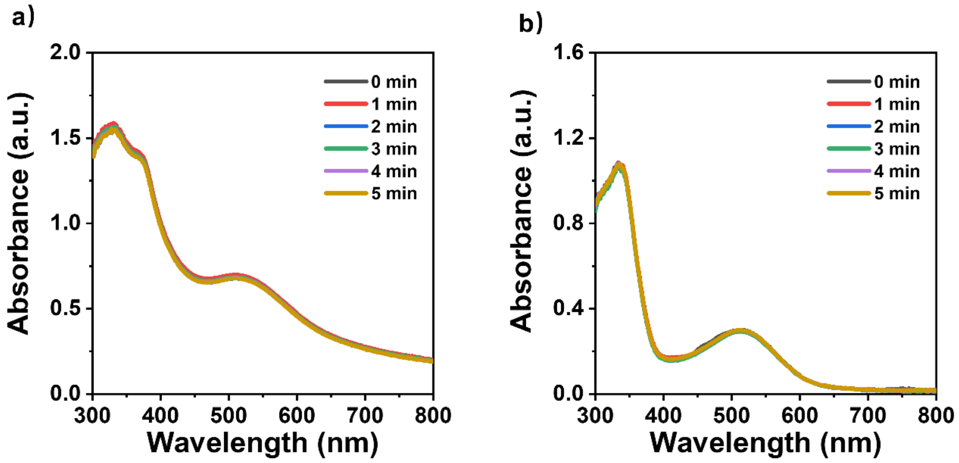

Figure S12. Stability of photosensitizers under light irradiation. The absorbance of a) Se6-NPs and b) Se5-NPs spectra under irradiation of white light (20 mW/cm^2). Concentrations of both Se6-NPs and Se5-NPs were 30 μM.

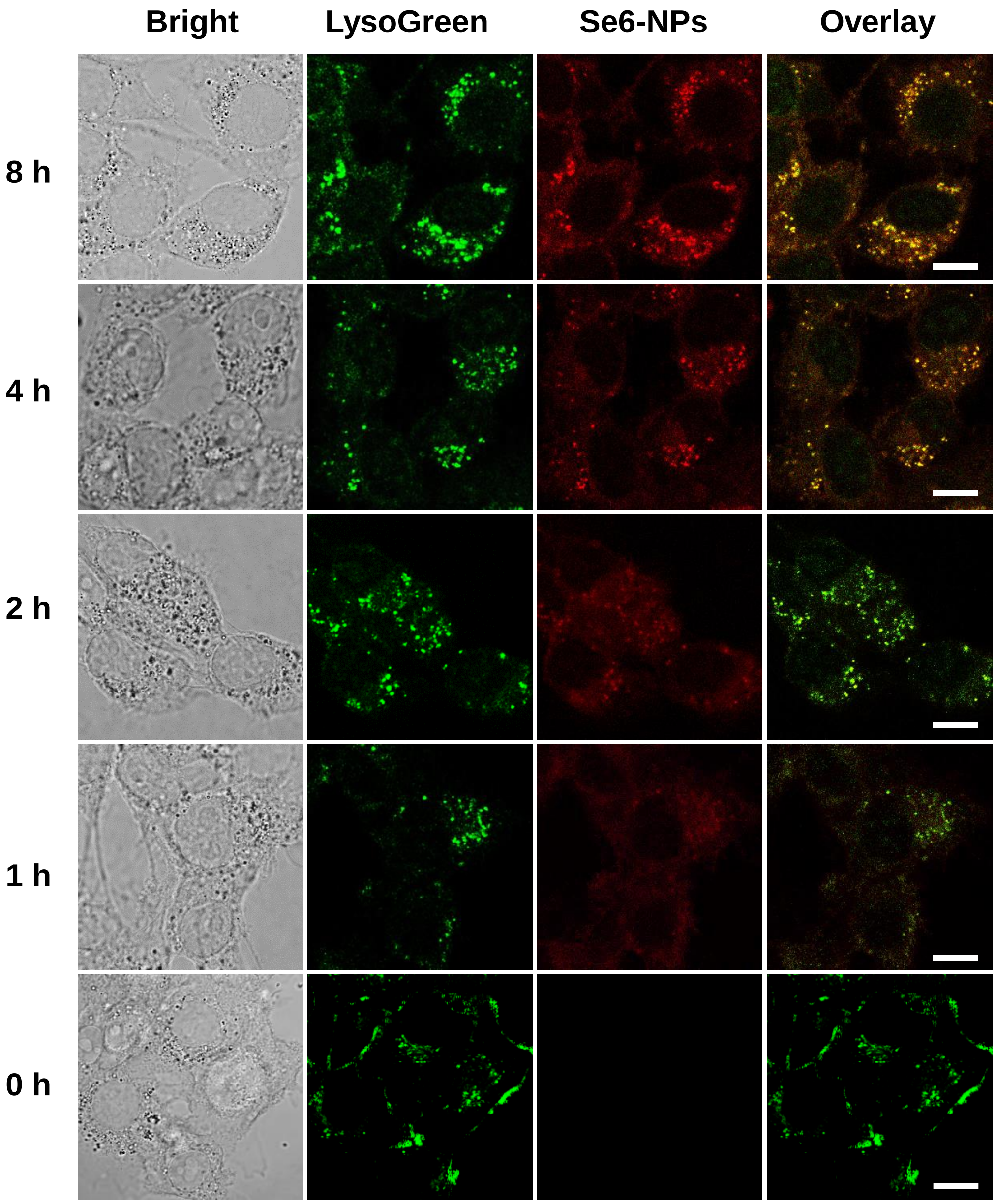

Figure S13. Confocal imagins of the location of Se6-NPs in cells over time: green fluorescence for lysosome probe (LysoGreen, emission: 510-540 nm), red fluorescence for Se6-NPs (emission : 650-690 nm), wavelength for excitation: 488 nm, scale bar was 10 μm.

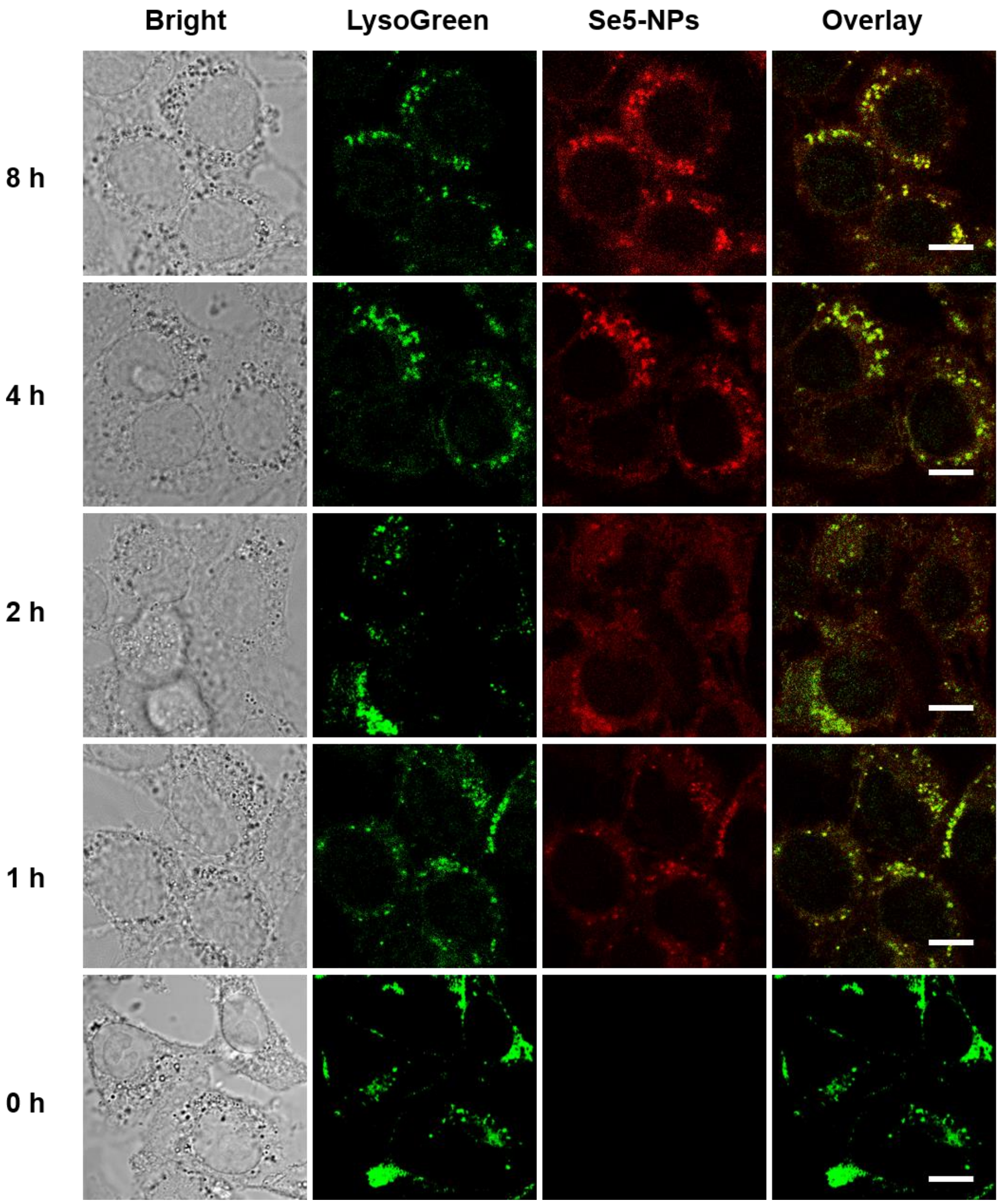

Figure S14. Confocal laser scanning microscopy (CLSM) images of the 4T1 cells stained with Se5-NPs and lysosome probe (LysoGreen) at different time points. The green fluorescence is LysoGreen, and the red fluorescence is Se5-NPs. Wavelengths for excitation (Ex) and emission (Em): Ex = 488 nm, Em = 510-540 nm for LysoGreen, Em = 650-690 nm for Se5-NPs, scale bar: 10 μm.

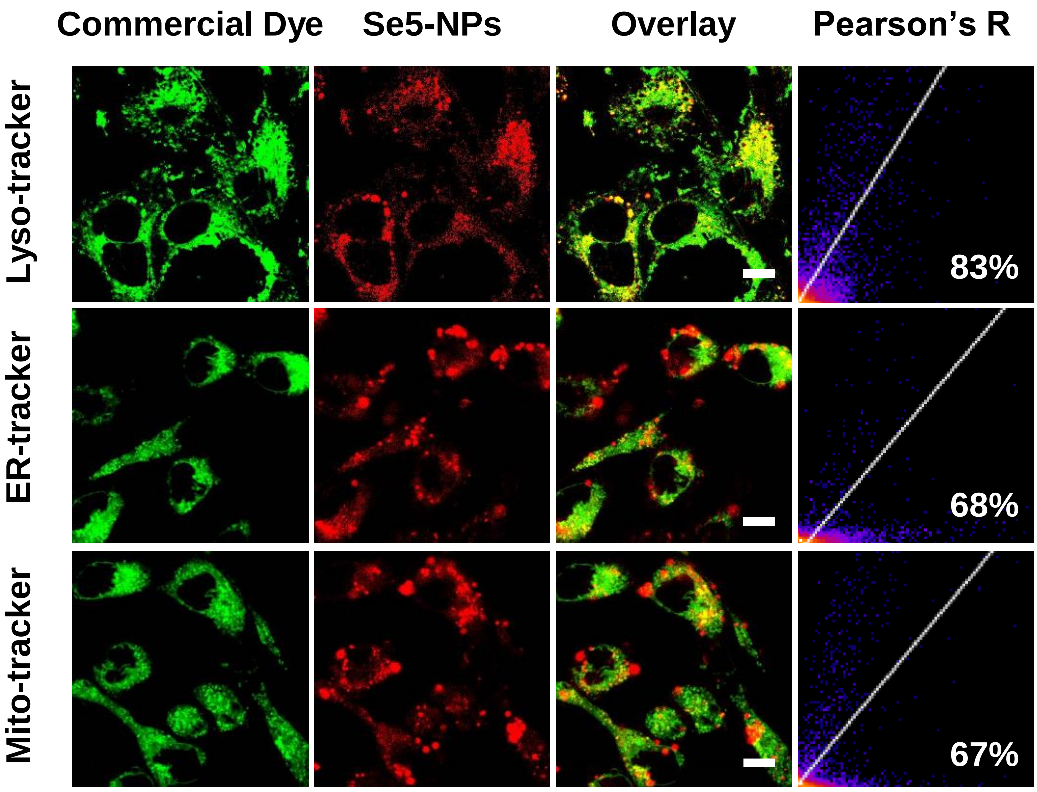

Figure S15. Colocalization images of 4T1 cells co-stained with Se5-NPs and different commercial dyes, including Lyso-Tracker Green, ER-Tracker Green, and Mito-Tracker Green. The excitation (Ex) and emission (Em) wavelengths for commercial dyes: Ex = 488 nm, Em = 510-540 nm; for Se5-NPs: Ex = 488 nm, Em = 650-690 nm; scale bar: 10 μm.

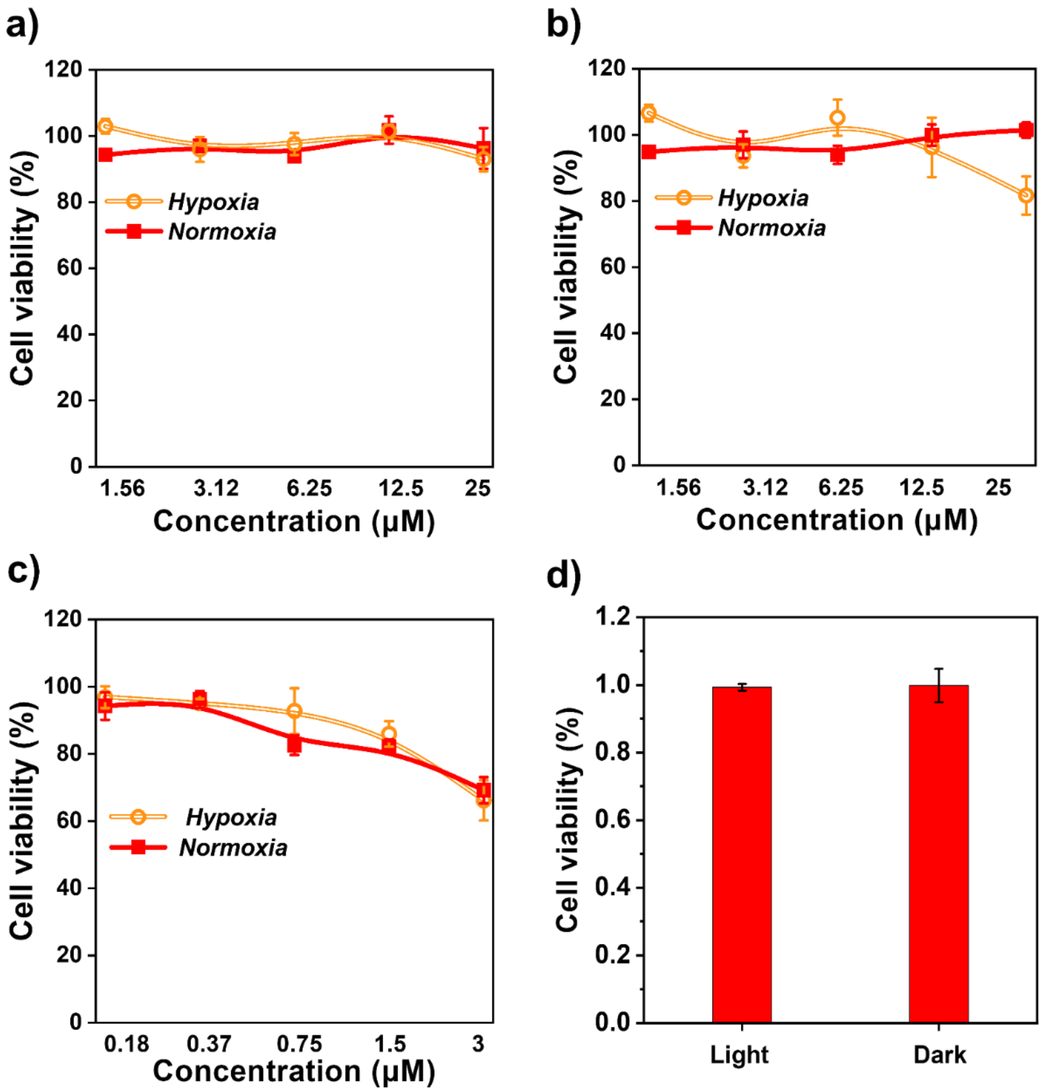

Figure S16. Cell viability of 4T1 cells in the dark incubated with a) Se6-NPs and b) Se5-NPs at various concentrations under normoxia or hypoxia conditions. c) Cell viability of 4T1 cells incubated with Se5-NPs at various concentrations upon irradiation of 20 mW cm^2 for 30 min under normoxia or hypoxia conditions. d) Cell viability of 4T1 cells under irradiation with white light of 20 mW/cm^2 for 30 min or in the dark. (n = 6, mean ± SD).

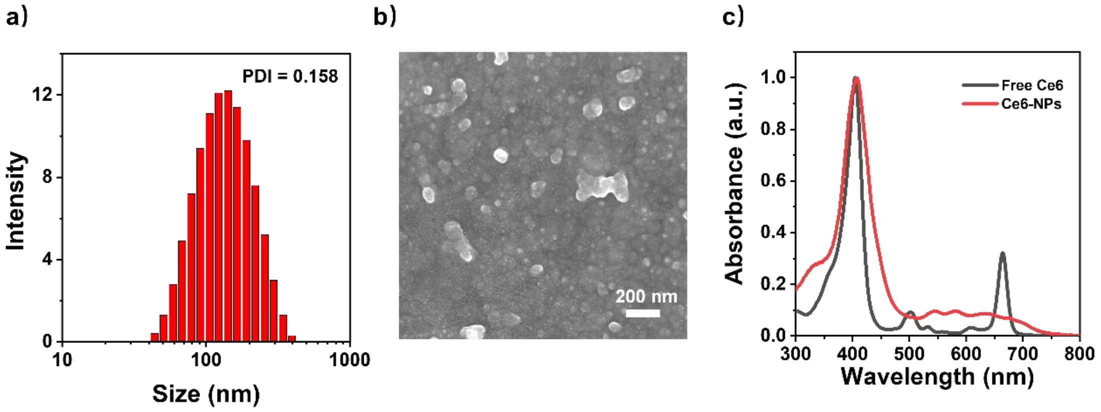

Figure S17. a) Particle size distribution and b) SEM image of Ce6-NPs. Scale bar in SEM image: 200 nm. c) UV spectra of free Ce6 in DMSO and Ce6-NPs in water, concentration: 10 μM. The PDI of nanoparticles was also inset in a).

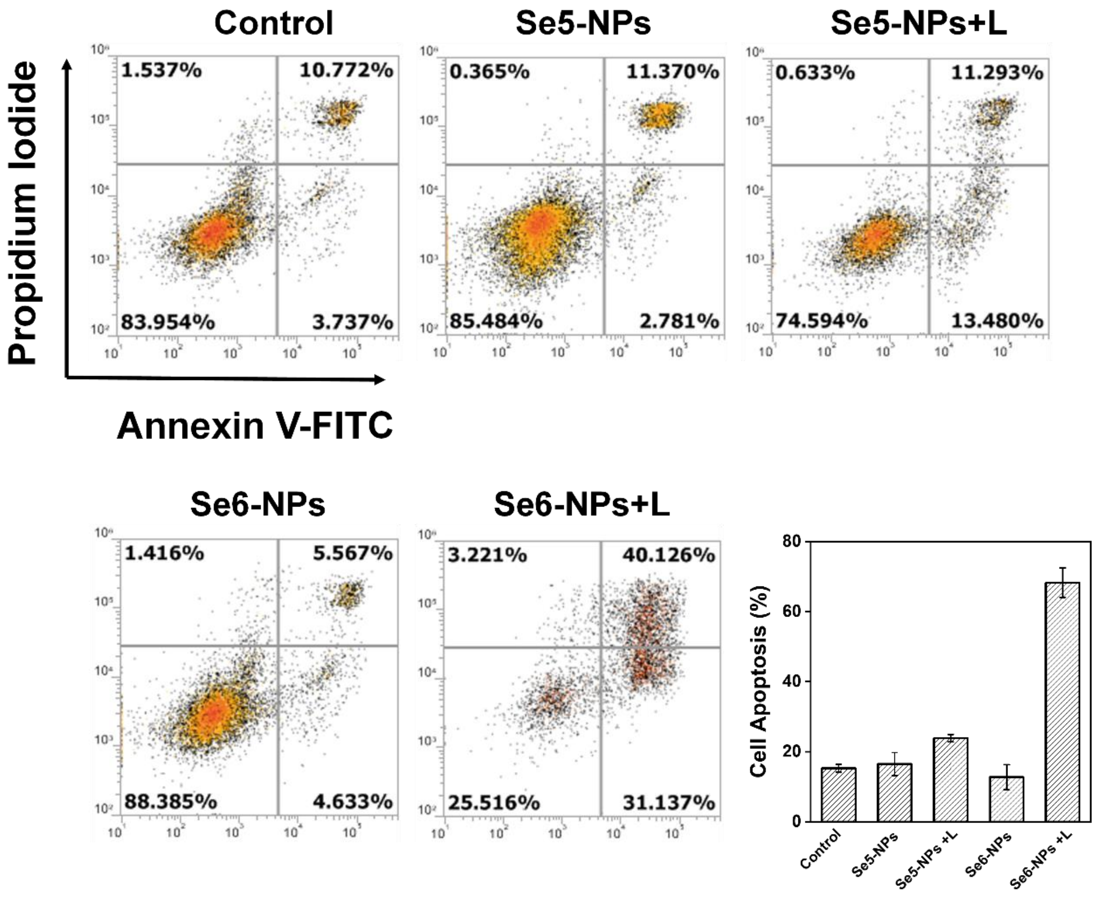

Figure S18. Apoptosis analysis using flow cytometry toward 4T1 cells after different treatments in various groups. Percentages of cell apoptosis for different groups calculated after PDT treatments were inset.

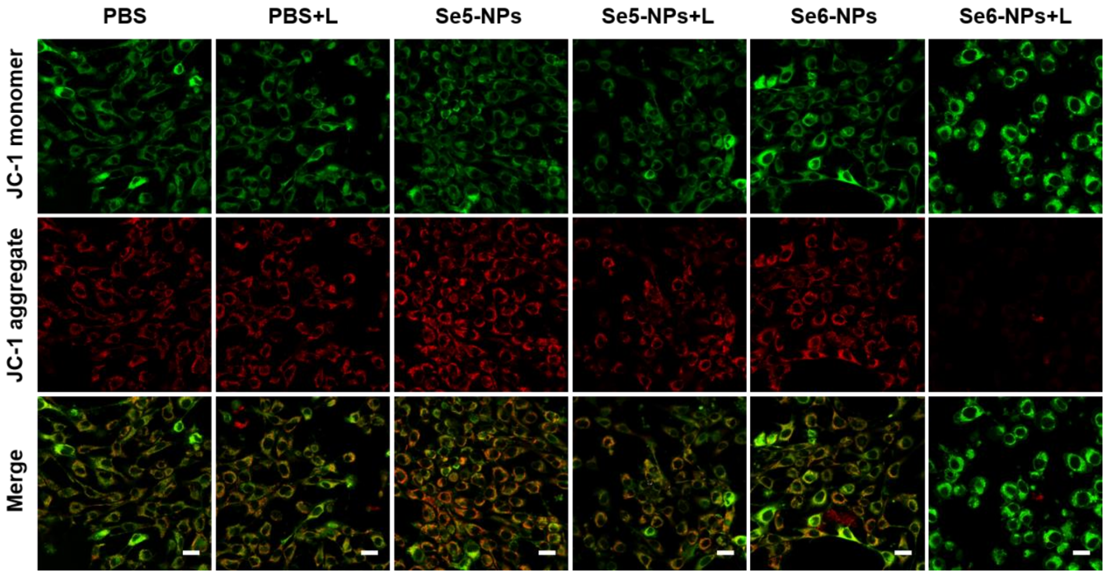

Figure S19. In vitro characterization about the damage of mitochondria on 4T1 cells in various groups by JC-1 fluorescent sensor with or without treatments upon irradiation of 20 mW cm^2 for 30 min. Scale bar: 20 μm; concentrations of Se6- and Se5- NPs: 3 μM; excited wavelength for both JC-1 monomer and aggregates: 488 nm; collected range of fluorescence for JC-1 monomer and aggregates: 510-540 nm and 580-610 nm, respectively.

As shown in Figure S19, the bright red fluorescence of JC-1 aggregates on surfaces of mitochondria became weak and almost disappeared in groups of Se5- NPs + light (L) and Se6-NPs + L, respectively. These differences on fluorescence compared to the other groups revealed the damage of mitochondria by ROS generated from Se5- and Se6-NPs.

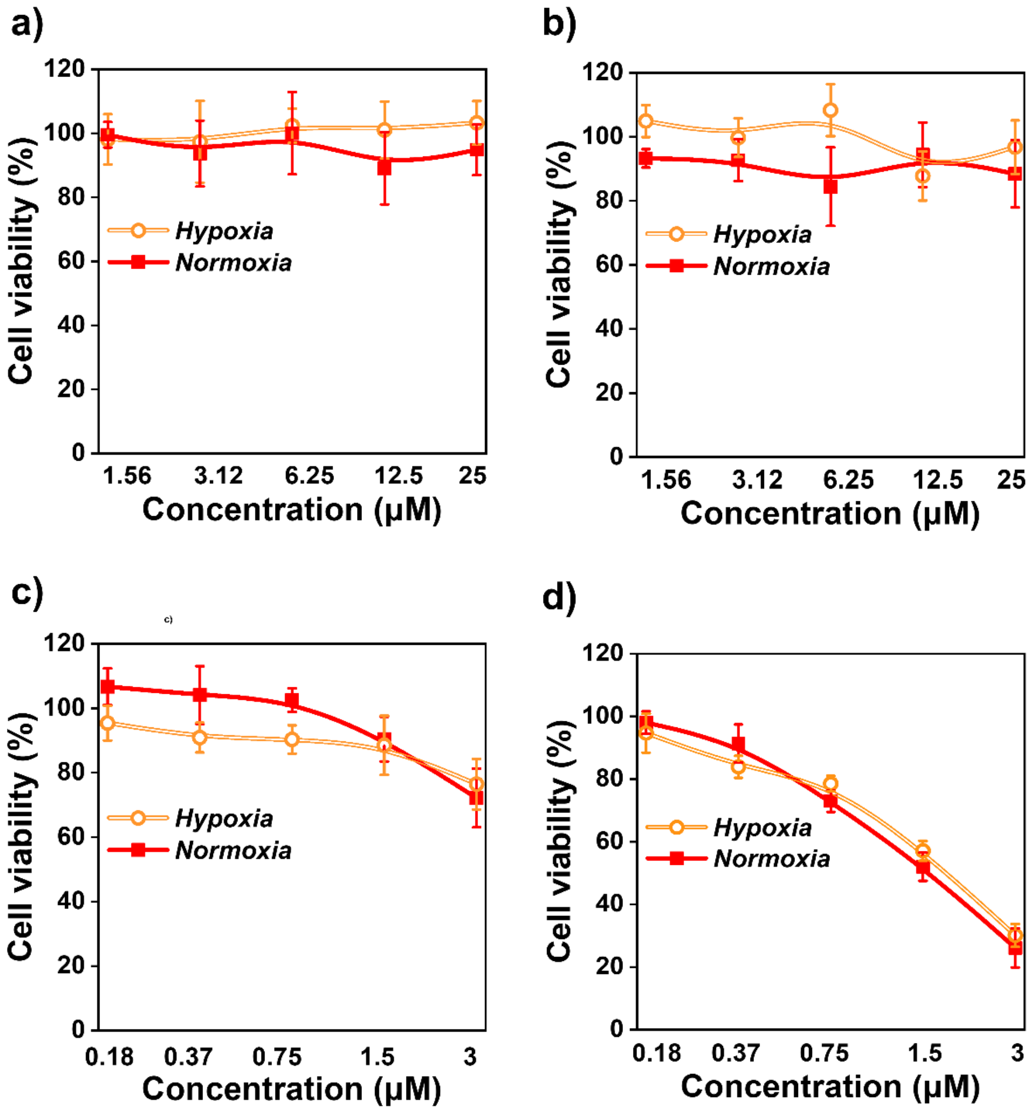

Figure S20. Cell viability of RM-1 cells in the dark incubated with a) Se6-NPs and b) Se5-NPs at various concentrations under normoxia or hypoxia conditions. Cell viability of RM-1 cells incubated with c) Se5-NPs and d) Se6-NPs at various concentrations upon irradiation of 20 mW cm^2 for 30 min under normoxia or hypoxia conditions. (n = 6, mean ± SD).

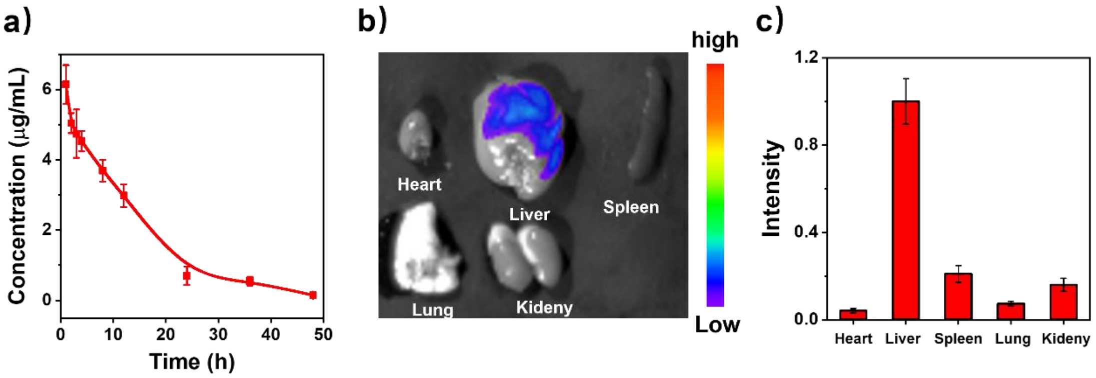

Figure S21. Pharmacokinetic investigation of Se6-NPs. a) Fluorescence intensity of Se6-NPs in blood of the mice injected of Se6-NPs. b) Ex vivo fluorescence images of major organs and e) their relative fluorescence intensity of Se6-NPs inside after 48 hours of tail vein injection. Wavelengths for excitation and emission: 520 and 670 nm, respectively. Concentration of Se6-NPs: 100 μM in 100 μL. (n = 3, mean ± SD).

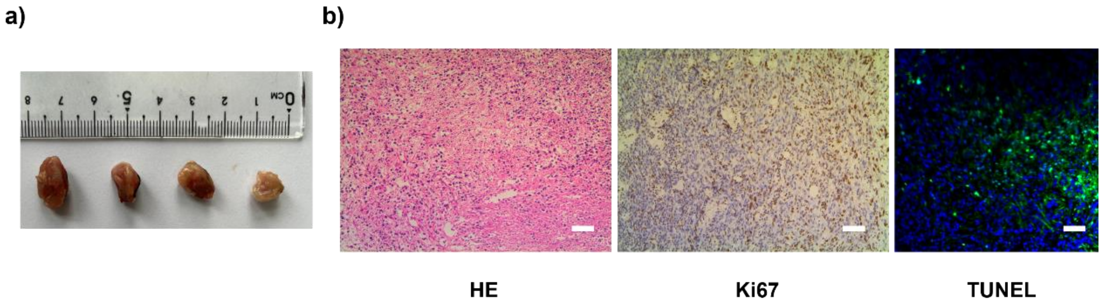

Figure S22. In vivo PDT treatment characteristic of Ce6-NPs on 4T1-tumor-bearing mice. a) Photos of tumor in vitro at day 21 after various treatments. b) Images of HE, Ki67 and TUNEL staining analysis of tumor slices. Scale bars: 100 μm; treatments: PBS + Ce6-NPs + L (Group 5).

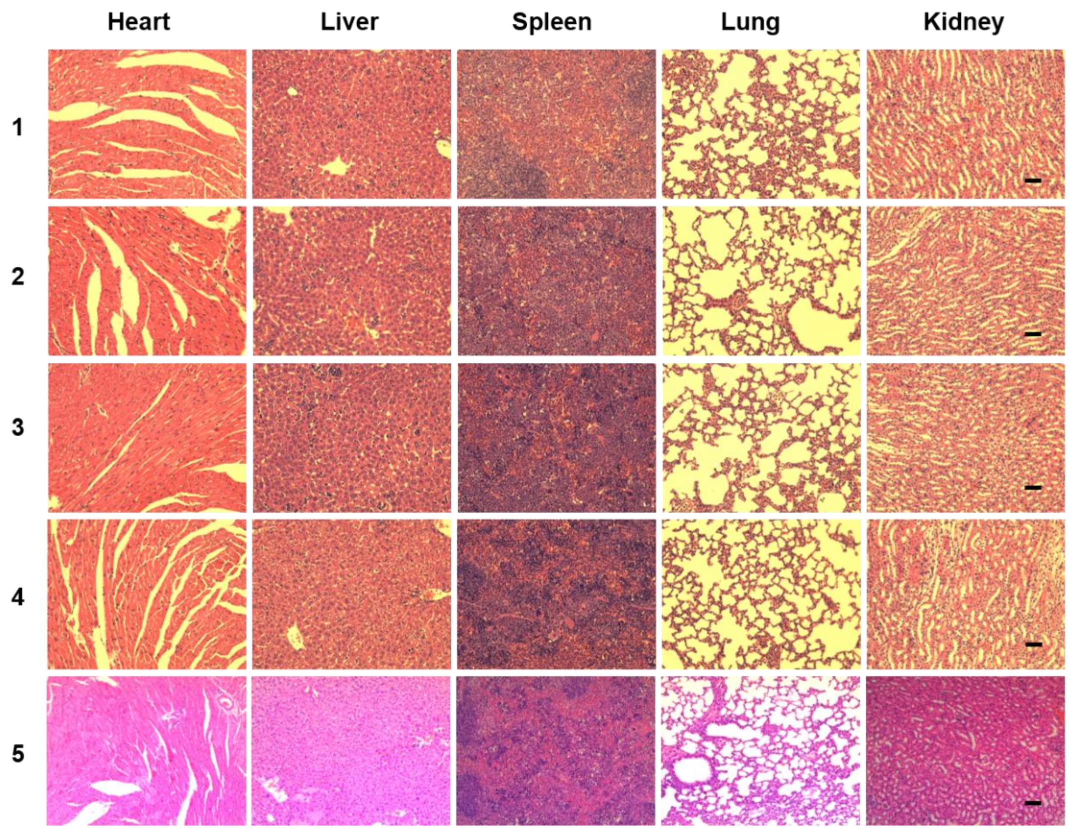

Figure S23. HE images for slices of main organs of these mice in various groups: heart, liver, spleen, lung, and kidney after treatments. Various treatments: PBS for Group 1, PBS + Light (L) for Group 2, PBS + Se6-NPs for Group 3, and PBS + Se6-NPs + L for Group 4, PBS + Ce6-NPs + L for Group 5; scale bar: 100 μm.

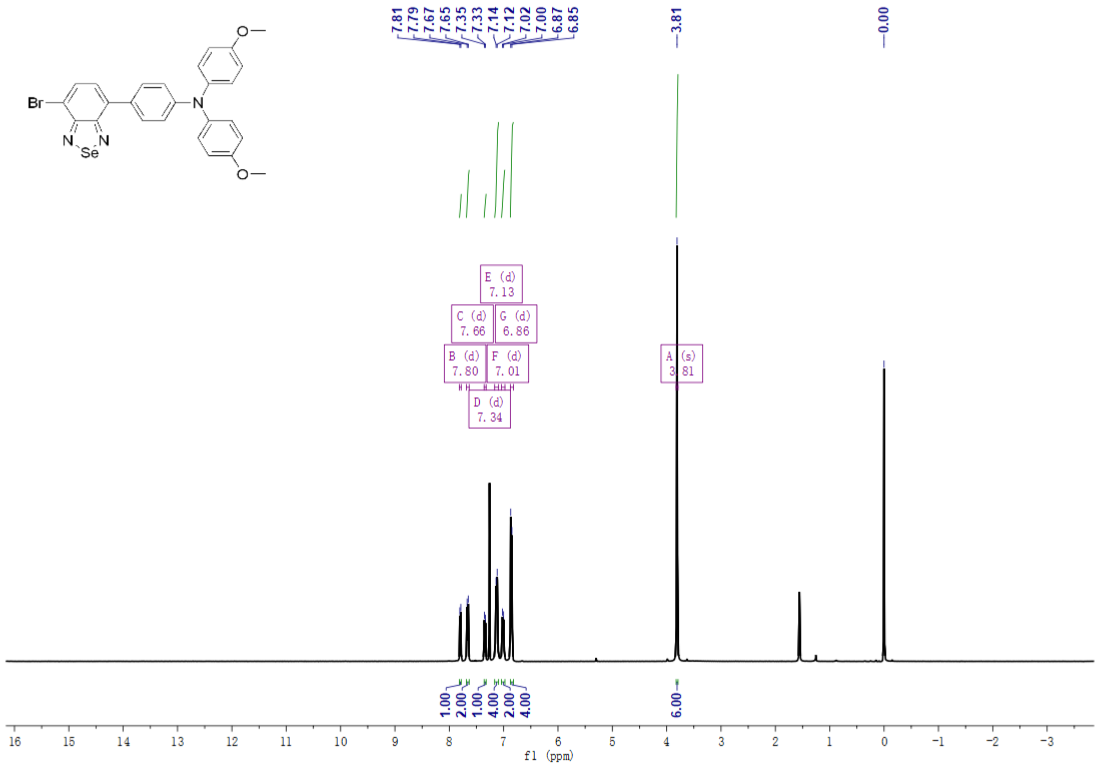

Figure S24. ^{1}H NMR spectrum of Se6 (400 MHz, $CDCl_3$, 298 K).

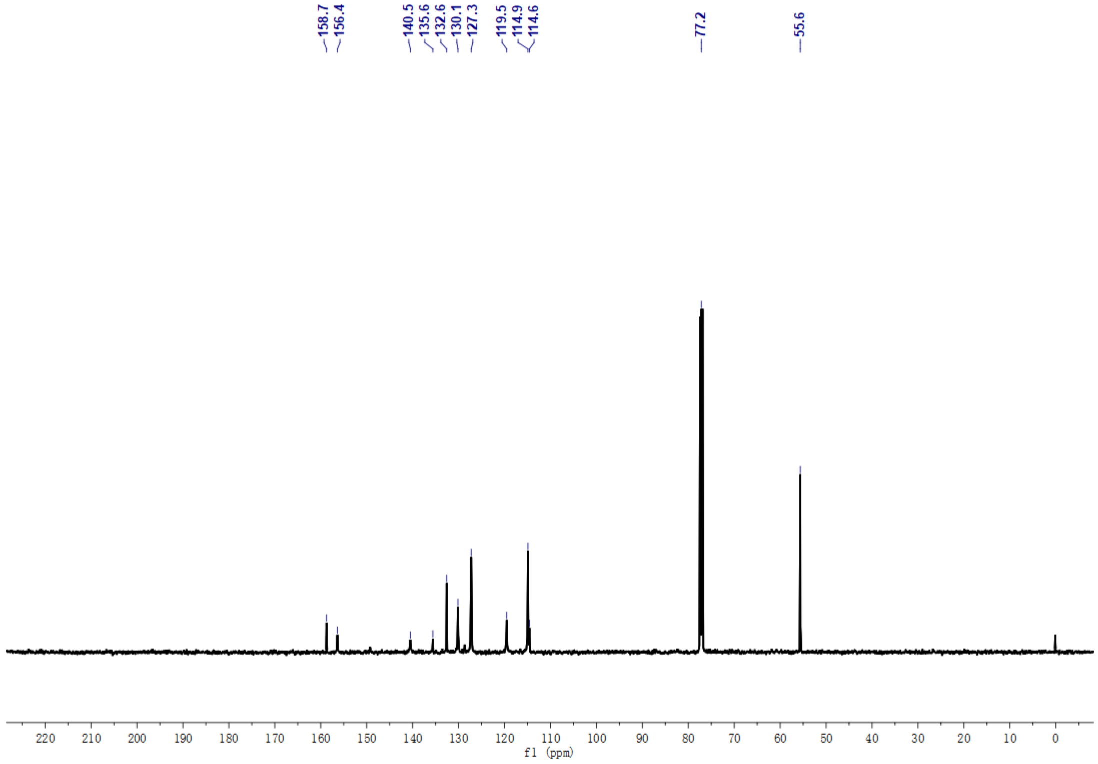

Figure S25. ^{13}C NMR spectrum of Se6 (100 MHz, $CDCl_3$, 298 K).

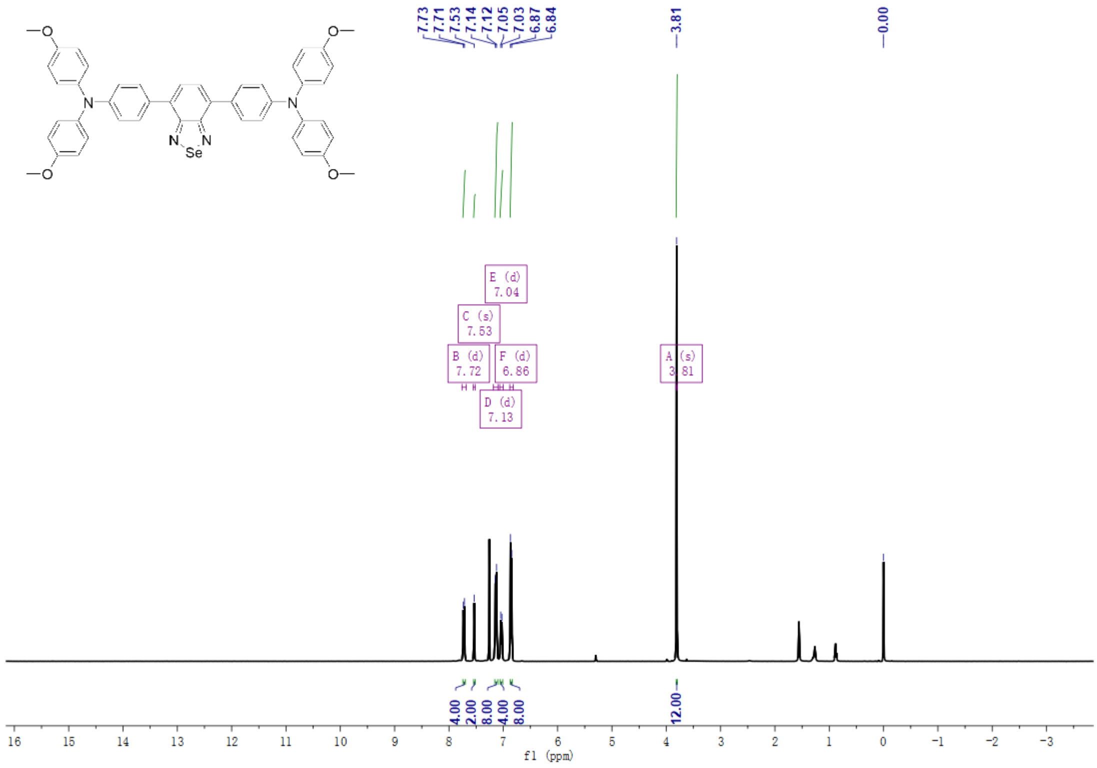

Figure S26. ^{1}H NMR spectrum of Se5 (400 MHz, $CDCl_3$, 298 K).

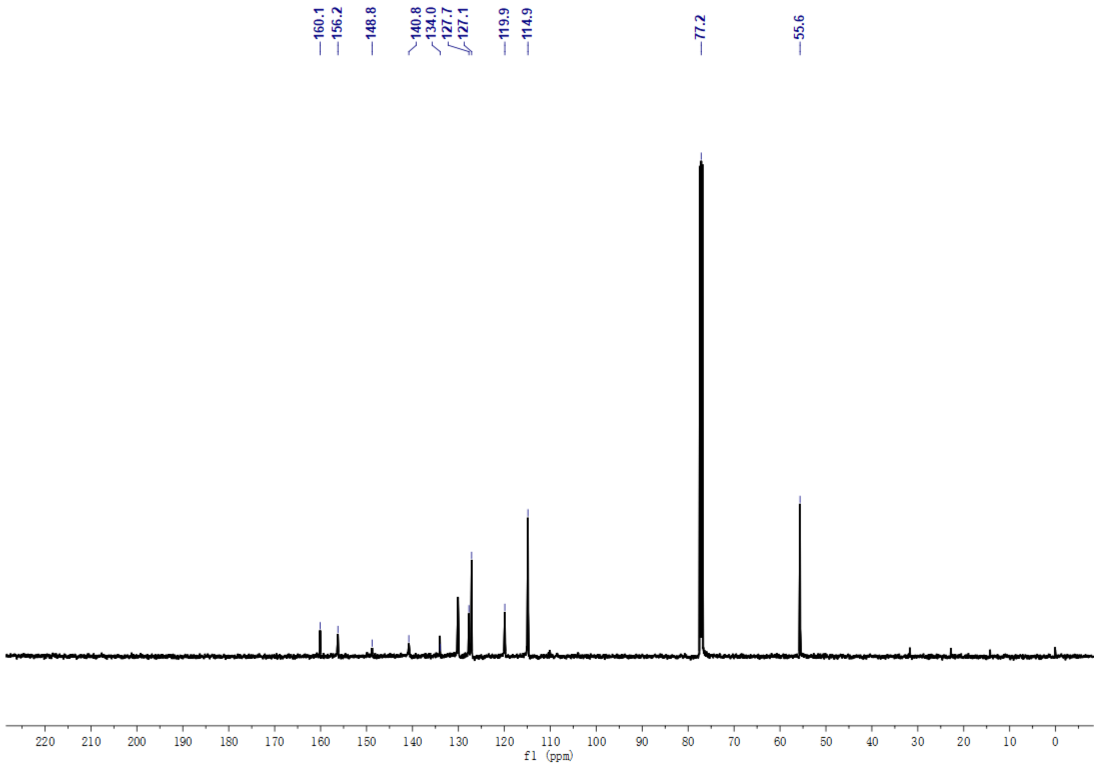

Figure S27. ^{13}C NMR spectrum of Se5 (100 MHz, $CDCl_3$, 298 K).

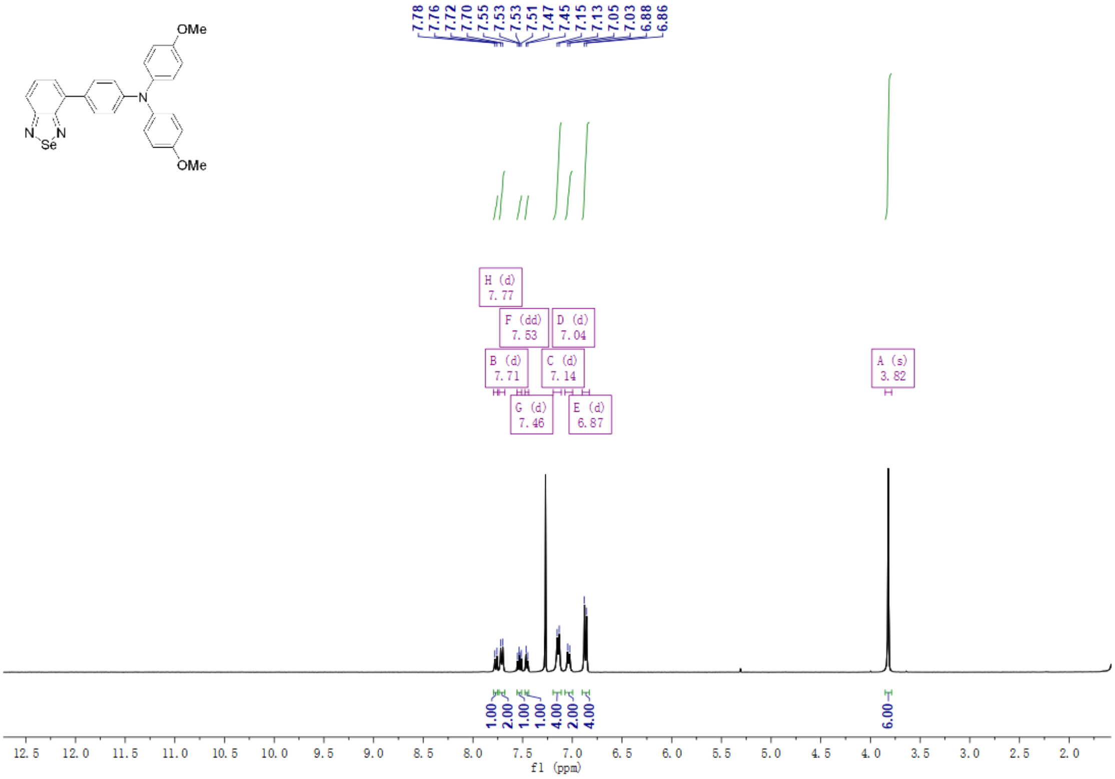

Figure S28. ^{1}H NMR spectrum of SeH (400 MHz, $CDCl_3$, 298 K).

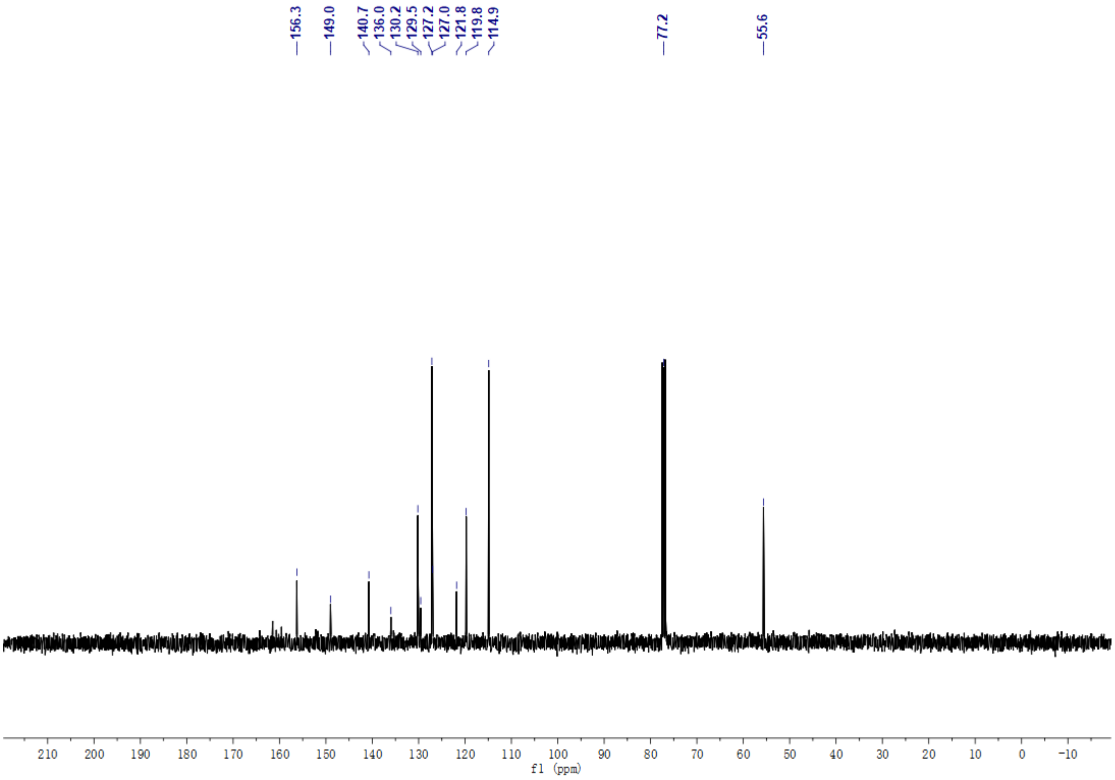

Figure S29. ^{13}C NMR spectrum of SeH (100 MHz, $CDCl_3$, 298 K).

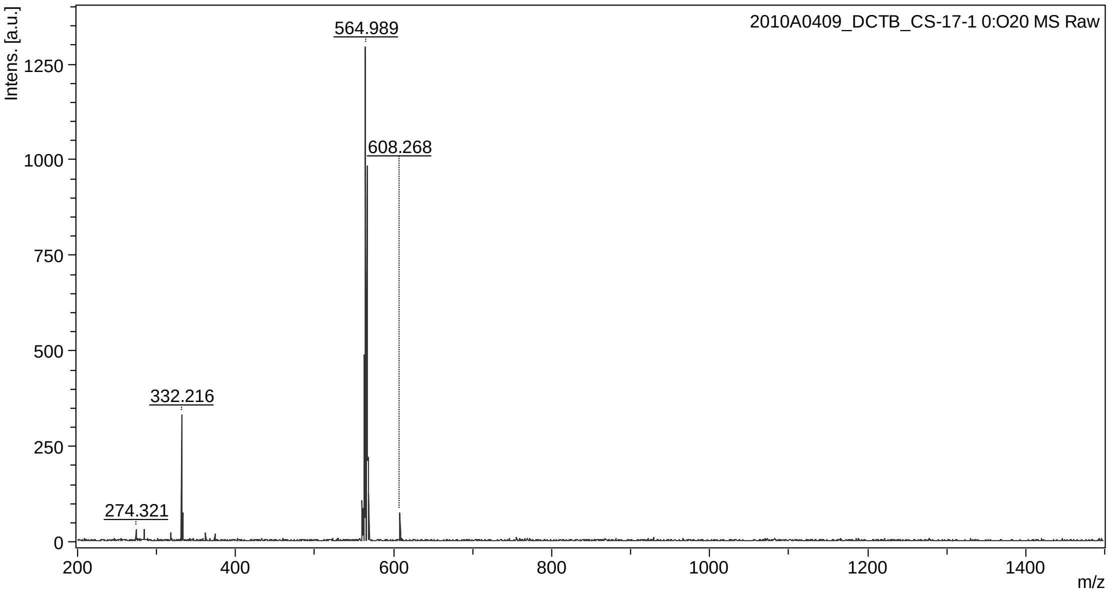

Figure S30. MALDI-TOF-MS spectrum of Se6.

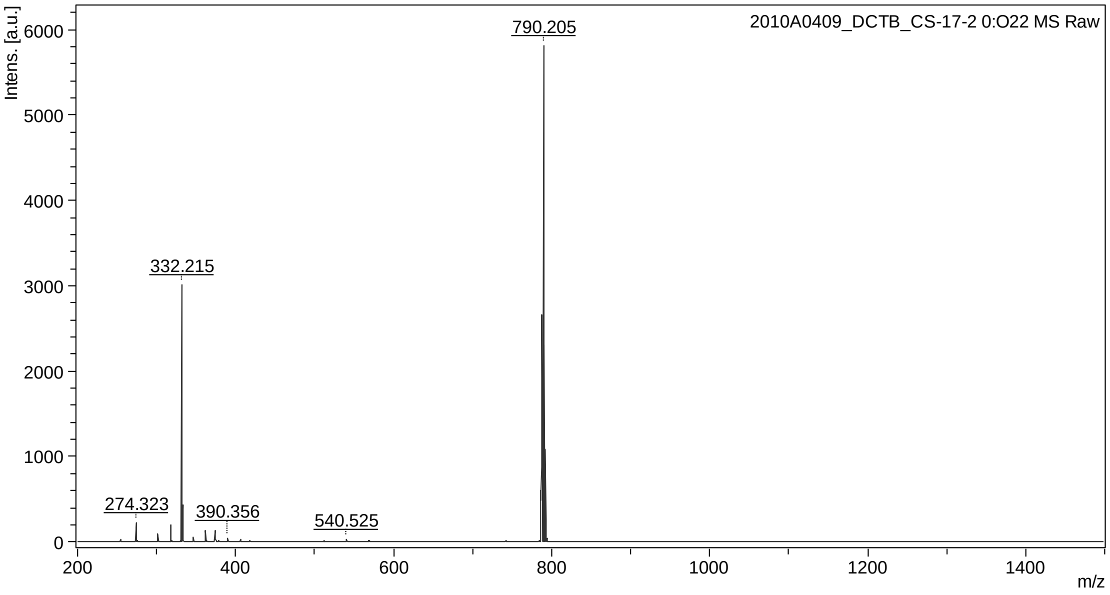

Figure S31. MALDI-TOF-MS spectrum of Se5.

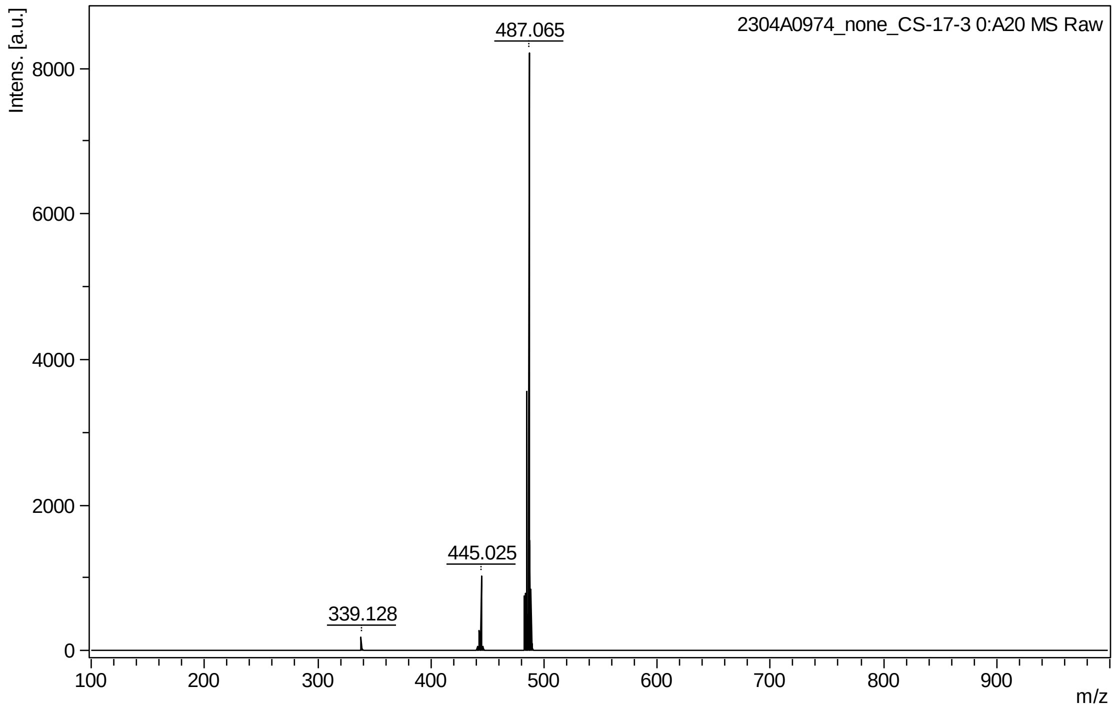

Figure S32. MALDI-TOF-MS spectrum of SeH.

Table S1 The experimental and calculated values of MOs for Se6 and Se5, respectively.

**Comp.**	**Experimental data / eV**		**Calculated data / eV**	
	HOMO	LOMO	HOMO	LOMO
Se6	-5.24	-3.06	-4.98	-2.72
Se5	-5.08	-2.90	-4.68	-2.14

[1] R. Xu, D.Dang, Z.Wang, Y. Zhou, Y. Xu, Y. Zhao, X. Wang, Z. Yang, L. Meng, *Chem. Sci*. **2022**, *13*, 1270.

[2] W. Zhang, Q. Li, Q. Zhang, Y. Lu, H. Lu, W. Wang, X. Zhao, X. Wang, *Inorg. Chem.* **2016,** *55*, 1005.
